# Targeting sphingolipid metabolism with the sphingosine kinase inhibitor SKI-II overcomes hypoxia-induced chemotherapy resistance in glioblastoma cells: effects on cell death, self-renewal, and invasion

**DOI:** 10.1186/s12885-023-11271-w

**Published:** 2023-08-16

**Authors:** Nadia Sousa, Carsten Geiß, Laura Bindila, Ingo Lieberwirth, Ella Kim, Anne Régnier-Vigouroux

**Affiliations:** 1 Institute of Developmental Biology & Neurobiology, Johannes Gutenberg University Mainz, Mainz, Germany; 2 Clinical Lipidomics Unit, Institute of Physiological Chemistry, Medical University Mainz, Mainz, Germany; 3 Max Planck Institute for Polymer Research, Mainz, Germany; 4 Department of Neurosurgery, Medical University of Mainz, Mainz, Germany

**Keywords:** Hypoxia, Glioblastoma therapy, Drug combination, Temozolomide, SKI-II, Sphingolipids, Invasion, Glioblastoma stem cells, Cell death

## Abstract

**Background:**

Glioblastoma patients commonly develop resistance to temozolomide chemotherapy. Hypoxia, which supports chemotherapy resistance, favors the expansion of glioblastoma stem cells (GSC), contributing to tumor relapse. Because of a deregulated sphingolipid metabolism, glioblastoma tissues contain high levels of the pro-survival sphingosine-1-phosphate and low levels of the pro-apoptotic ceramide. The latter can be metabolized to sphingosine-1-phosphate by sphingosine kinase (SK) 1 that is overexpressed in glioblastoma. The small molecule SKI-II inhibits SK and dihydroceramide desaturase 1, which converts dihydroceramide to ceramide. We previously reported that SKI-II combined with temozolomide induces caspase-dependent cell death, preceded by dihydrosphingolipids accumulation and autophagy in normoxia. In the present study, we investigated the effects of a low-dose combination of temozolomide and SKI-II under normoxia and hypoxia in glioblastoma cells and patient-derived GCSs.

**Methods:**

Drug synergism was analyzed with the Chou-Talalay Combination Index method. Dose–effect curves of each drug were determined with the Sulforhodamine B colorimetric assay. Cell death mechanisms and autophagy were analyzed by immunofluorescence, flow cytometry and western blot; sphingolipid metabolism alterations by mass spectrometry and gene expression analysis. GSCs self-renewal capacity was determined using extreme limiting dilution assays and invasion of glioblastoma cells using a 3D spheroid model.

**Results:**

Temozolomide resistance of glioblastoma cells was increased under hypoxia. However, combination of temozolomide (48 µM) with SKI-II (2.66 µM) synergistically inhibited glioblastoma cell growth and potentiated glioblastoma cell death relative to single treatments under hypoxia. This low-dose combination did not induce dihydrosphingolipids accumulation, but a decrease in ceramide and its metabolites. It induced oxidative and endoplasmic reticulum stress and triggered caspase-independent cell death. It impaired the self-renewal capacity of temozolomide-resistant GSCs, especially under hypoxia. Furthermore, it decreased invasion of glioblastoma cell spheroids.

**Conclusions:**

This in vitro study provides novel insights on the links between sphingolipid metabolism and invasion, a hallmark of cancer, and cancer stem cells, key drivers of cancer. It demonstrates the therapeutic potential of approaches that combine modulation of sphingolipid metabolism with first-line agent temozolomide in overcoming tumor growth and relapse by reducing hypoxia-induced resistance to chemotherapy and by targeting both differentiated and stem glioblastoma cells.

**Supplementary Information:**

The online version contains supplementary material available at 10.1186/s12885-023-11271-w.

## Background

Glioblastoma (GB) is the most common and malignant form of primary brain tumors. The standard treatment for newly diagnosed cases is surgical resection followed by radiotherapy plus concomitant and adjuvant temozolomide (TMZ) chemotherapy [1]. TMZ primarily induces cytotoxicity through methylation of guanine at position O6 (O6-methylguanine) [2], triggering G2/M cell cycle arrest and, eventually, apoptosis. However, the enzyme O6-methylguanine-DNA methyltransferase (MGMT) is able to repair this DNA adduct [3]. Intrinsic and acquired resistance to TMZ contributes to inevitable tumor recurrence and a short median survival of patients [4]. Currently, there is no established treatment for recurrent GB [5]. Tumor hypoxia, a hallmark of GB and other solid tumors, promotes tumor cell invasion into the brain parenchyma and contributes to therapy resistance [6]. Moreover, hypoxia and TMZ favor the expansion of the glioblastoma stem cell (GSC) population, which are undifferentiated cancer cells with unlimited self-renewal capability and propensity to promote intratumoral heterogeneity [7, 8]. GSCs are considered important drivers of tumor relapse after initial treatment [9]. Thus, prevention of GB recurrence requires the combined targeting of differentiated tumor cells and GSCs. Sphingolipids are involved in a variety of biological functions. Regulation of their metabolism is thus essential for cell homeostasis and fate and, as such, it has evolved as a very attractive therapeutic target [10]. Sphingosine kinases (SK) 1 and 2, are enzymes controlling the balance between the sphingolipids sphingosine-1-phosphate (S1P) and ceramide. Ceramide is catabolized by ceramidases into sphingosine which can be further phosphorylated to generate S1P [10]. S1P can be exported into the extracellular milieu, stimulate five G-protein-coupled receptors (S1PR1-5) and regulate intracellular signaling pathways involved in proliferation, invasion, angiogenesis, and stem cell properties [11]. In contrast, ceramide is known for its pro-apoptotic activities [12]. Ceramide can be generated from sphingosine via ceramide synthases, but also synthetized in the de novo pathway from dihydroceramide via dihydroceramide desaturase 1 (DES1), or generated from the hydrolysis of sphingomyelin via sphingomyelinase. Glioblastoma tissue specimens contain higher S1P and lower ceramide levels relative to healthy tissue, and high SK1 expression correlated with a shorter survival time of GB patients [13]. Various chemotherapeutic agents have been shown to trigger tumor cell death by favoring the production of ceramide [14]. However, in the case of glioblastoma, this increased ceramide production might be counteracted by SK overexpression, leading to conversion of ceramide into S1P. Blocking SK might prevent this conversion and support TMZ efficacy. The dual sphingosine kinase inhibitor SKI-II is orally bioavailable and showed significant in vivo antitumor activity without displaying toxicity [15]. Although first described as a sphingosine kinase inhibitor, it was later demonstrated to have off target effects on DES1 [16]. Reported inhibition constants (Ki) indicate a stronger effect of SKI-II on DES1 (Ki = 0.3 µM) than on SK1 (Ki = 16 µM) [17]. We have previously shown that 10 µM SKI-II combined with 500 µM TMZ induced caspase-dependent cell death in GB cells, preceded by accumulation of dihydrosphingosine and dihydroceramide, oxidative stress, endoplasmic reticulum (ER) stress, and autophagy. In that experimental setup, dihydrosphingolipids, and not ceramide, were the pro-apoptotic sphingolipids, reflecting the inhibitory activities of SKI-II towards both SK1 and DES1 [18]. Other studies have as well demonstrated dihydroceramide to be involved in ER stress, autophagy, and proliferation [19].

The raising interest in modulating the sphingolipid metabolism and specifically the sphingosine/S1P axis for therapeutic purposes is reflected by the increased number of SK1 and SK2 inhibitors developed in the last years. Whereas some inhibitors are tested in clinical trials (e.g. the SK2 inhibitor ABC294640; see reference in [20], most SK inhibitors (including SKI-II and more recently described SK1 inhibitors) are still in the preclinical testing phase [20]. This slow translation to the clinic indicates that we need to deepen our knowledge of the effects these inhibitors exert on biological processes and on sphingolipid metabolism for a better evaluation of their therapeutic value and affected pathway(s). In the present study, we further investigated the therapeutic potential of SKI-II combined with TMZ at clinically relevant doses, based on the concentrations detected in the serum and tumor of patients [21, 22], and we compared the effects of the combination under normoxic (21% O_2_) and hypoxic (3% O_2_) conditions. We report that a low-dose combination (48 µM TMZ + 2.66 µM SKI-II) has potent anti-tumor activities under hypoxia, such as cell growth inhibition and cell death induction, self-renewal impairment of TMZ-resistant GSCs, and reduction of the invasion capacity of recurrent GB cell-derived spheroids.

## Methods

### Reagents

Temozolomide, SKI-II, Camptothecin, Z-VAD-FMK, Sulforhodamine B, FCCP and FeTPPS were purchased from Sigma-Aldrich. All drugs were dissolved in DMSO as stock solutions and kept at -20°C. Working dilutions of all drugs were performed in culture media immediately before each experiment. AnnexinV-FITC and 10X Annexin Binding Buffer were purchased from Dianova. MitoStatus TMRE was purchased from BD Pharmingen.

### Cell culture

The low-passage-number human glioblastoma cell line NCH82 was generated at the Department of Neurosurgery, Heidelberg University Hospital (Heidelberg, Germany) [23]. NCH82 cells were cultured in complete DMEM (cDMEM) [Dulbecco's Modified Eagle Medium (Sigma),10% (vol/vol) fetal calf serum (Biochrom), 2 mM L-glutamine (Life Technologies), gentamicin (50 μg/ml) (GIBCO)]. NCH82 cells were kept in culture for no longer than six passages after thawing.

The human glioblastoma stem-like cell (GSC) line No. 1080 was generated at the University Medical Center Göttingen (Göttingen, Germany) [24]. GSCs were cultured as spheres in NeuroBasal medium (GIBCO) supplemented with the B27 component (Invitrogen Life technologies), fibroblast growth factor (FGF) (Peprotech, 10 ng/ml) and epidermal growth factor (EGF) (Peprotech, 20 ng/ml). 1080 GSCs were selected with 100 µM TMZ (hereafter referred as TMZ-1080), and the respective DMSO control (hereafter referred as DMSO-1080).

The human glioblastoma cell line U3054MG (mesenchymal subtype) was acquired from the HGCC (Human Glioblastoma Cell Culture Resource) where it was established from a GB that recurred after the patient had undergone surgery and radiotherapy (50.4 Gy in total) [25]. Cells were cultured in laminin-coated Primaria dishes (Corning) in Neurobasal and DMEM/F12 media (1:1 mix) containing N2 and B27 supplements (Invitrogen) and human recombinant FGF and EGF (10 ng/ml, Peprotech).

All cells were cultured at 37°C, 5% CO_2_ under normoxic (21% O_2_) conditions. All treatments were performed in culture medium containing 5% FCS.

Cell cultures were regularly tested for mycoplasma contamination (MycoAlert™, Lonza Bioscience). The NCH82 and 1080 cell lines were authenticated via Single Nucleotide Polymorphism (SNP)-profiling.

### Sulforhodamine B assay

The sulforhodamine B (SRB) colorimetric assay was used to determine the dose–effect curves of each drug alone and assess the effect of drug combinations. NCH82 cells were seeded in 96-well plates, allowed to attach for 24 h and then treated with each drug and vehicle control (DMSO) in quadruplicates. The cells were further incubated for 5 days at 21% and 3% O_2_ in dedicated incubators. The SRB assay was performed as described [26]. The absorbance (OD) of each well was read at 510 nm on a microplate reader (TECAN Infinite M200 PRO). Background absorbance was subtracted from all samples. Percentage of control cell growth was calculated using the formula: % of control cell growth = [(mean OD _sample_ at d5 – mean OD at d0) / (mean OD _control_ at d5 – mean OD at d0)] × 100.

### Propidium iodide and Annexin V staining of NCH82 cells and GSC cells

Flow cytometry was used to assess drug-induced cell death by performing staining with propidium iodide (PI) and Annexin V-FITC (Dianova). NCH82 cells were seeded in 12-well plates and treated accordingly in duplicates 24 h later. After 3 and 5 days of incubation at 21% and 3% O_2_, adherent and floating cells were collected and washed twice with 1X Annexin V binding buffer (Dianova). Then, 5 µl of Annexin V-FITC and 1 µg/ml of PI were added to 100 µl of cell suspension and incubated for 15 min in the dark at room temperature. Finally, cells were analyzed with the Attune NxT Flow Cytometer (Thermo Fisher Scientific) at a sample flow rate of 100 µl/min. Flow cytometry data were analyzed with the software FlowJo. The following gating strategy was applied: doublets discrimination (SSC-A vs SSC-H); selection of the population of interest/debris elimination (SSC-H vs FSC-H) and application of the two-color gating/quadrant (BL2-H vs BL1-H). Spheres of the DMSO-1080 and TMZ-1080 GSC cells were dissociated using Trypsin–EDTA 0,05% (Thermo Fischer Scientific) diluted at a 1:1 ratio in NeuroBasal medium, seeded in 12-well plates and treated accordingly in duplicates 24 h later. After 5 days of incubation at 21% O_2_ and 3% O_2_, adherent and floating cells were collected and processed for staining and analysis at the flow cytometer as described for the NCH82 cells.

### Drug combination analysis

The combination index (CI) theorem of Chou and Talalay was used to quantitatively determine synergism between combinations of TMZ and SKI-II [27–29]. A combination is considered to be synergistic when CI is below 1, antagonistic when CI is greater than 1, and to have an additive effect when CI equals 1. The CI was calculated via the third-generation software CompuSyn [30]. First, dose–effect curves of each drug alone were determined by exposing cells to twofold serial dilutions of TMZ and SKI-II for 5 days at 21% and 3% O_2_. The percentage of cell growth was measured using the SRB assay as previously described. This allowed to determine the parameters *D*_*m*_ and *m* for each drug, which are required for CI calculation. *D*_*m*_ is the dose required to achieve a 50% effect level (ED50), and *m* depicts the shape of the dose–effect curve (m = 1 hyperbolic; m > 1 sigmoidal; m < 1 negative sigmoidal). Additionally, the parameter *r* (linear correlation coefficient of the median effect plot) was obtained and refers to the conformity of the data to the mass-action law (for in vitro studies *r* > 0.95). Next, combinations of TMZ and SKI-II at non-constant ratios were tested for 5 days at 21% and 3% O_2_. Two-fold serial dilutions below and above the determined ED50 (21% O_2_) were performed for SKI-II, and for TMZ only below the ED50, since values above the ED50 (21% and 3% O_2_) are clinically irrelevant.

### In vitro extreme limiting dilution assay

GSCs (DMSO-1080 and TMZ-1080) were seeded in 24-well plates in complete NeuroBasal medium at a density of 10 cells/ml and, subsequently, serial diluted until the theoretical cell density of 0.3125 cells/ml. The number of wells used per cell density for each condition ([DMSO, TMZ, SKI-II and (TMZ + SKI-II)] in every independent experiment was the following: 6 wells for 10 cells/ml; 12 wells for 5 cells/ml; 18 wells for 2.5 cells/ml; 18 cells for 1.25 cells/ml; 12 wells for 0.625 cells/ml.; and 6 wells for 0.3125 cells/ml. Cells were treated 24 h later and further incubated for 4 weeks at 21% and 3% O_2._ After the incubation period, the number of wells containing at least one sphere (positive cultures) was registered for each dose (number of cells/well). The stem cell frequency was determined using the webtool ELDA provided by the Walter and Eliza Hall Institute Bioinformatics Division (https://bioinf.wehi.edu.au/software/elda/) [31].

### Spheroid invasion assay

U3054 spheroids were generated for 48 h in ultra-low attachment 96-well plates (CellCarrier Spheroid ULA 96-well Microplates, PerkinElmer) at a density of 8000 cells/40 µl/well/spheroid. Spheroids of approximately 400 µm diameter were embedded in a collagen mixture composed of 10X Minimum Essential Medium, 0.1 M NaOH, 3 mg/ml of PureCol® Bovine Collagen solution type I (Advanced Biomatrix) and respective treatment. After 1 h incubation at 37 °C, complete DMEM (5% FCS) containing the respective treatment was added on the surface of the polymerized collagen mixture and light microscopy images were collected from each spheroid (day 0). Embedded spheroids were further incubated at 21% O_2_ or 3% O_2_ and images were collected daily. These images were uploaded in the software Spheroid Analyzer from CLADIAC [32] which automatically determined the invaded area (including the core of the spheroid) for each day and calculated its relative size compared to day 0.

### Detection of activated caspase-3 in treated NCH82 cells and GSC cells

NCH82 cells seeded in 12-well plates were treated accordingly in duplicates 24 h later. After drug incubation time at 21% and 3% O_2_, adherent and floating cells were collected and washed twice with cold 1X PBS, resuspended in BD Cytofix/Cytoperm solution (BD Biosciences) and incubated 20 min on ice. Then, cells were washed twice with 1X BD Perm/Wash buffer (BD Biosciences) at room temperature and incubated for 30 min with 30 µl of FITC Rabbit Anti-Active Caspase-3 antibody (BD Biosciences) in 100 µl of 1X BD Perm/Wash buffer at room temperature. Finally, each sample was washed and resuspended in 1X BD Perm/Wash buffer and analyzed by flow cytometry. Flow cytometry data were analyzed via the software FlowJo. The following gating strategy was applied: doublets discrimination (SSC-A vs SSC-H); selection of the population of interest/debris elimination (SSC-H vs FSC-H) and application of one-color gating (BL1-H vs FSC-H).

Spheres of DMSO-1080 and TMZ-1080 GSC cells were dissociated using Trypsin–EDTA 0,05% diluted at a 1:1 ratio in NeuroBasal medium, plated on ornithin-coated glass coverslips and treated accordingly in triplicates 24 h later. After 5 days of incubation at 21% and at 3% O_2_, cells were fixed with 4% paraformaldehyde, washed with PBS, incubated in blocking solution (0.1% Triton X-100, 1% bovine serum albumin) and stained overnight at + 4 °C with a mix of antibodies specific for cleaved caspase-3 (Cell Signaling) and human nestin (Abcam). Secondary antibodies were goat α-mouse Alexa Fluor 488 (Invitrogen) and goat α-rabbit Alexa Fluor 555 (Invitrogen). Dapi staining was performed according to standard conditions. Stained cells were analyzed by using the BZ-X fluorescence microscope (Keyence Germany GbmH). For each condition (triplicate), 700 to 1100 cells were counted from a total of 7 to 9 regions. Percentage of cells positive for cleaved caspase-3 was determined by using the Image Tool software.

### Measurement of mitochondrial membrane potential

NCH82 cells were seeded in 12-well plates and treated accordingly in triplicates 24 h later. Cells were treated with (TMZ + SKI-II) for 24 and 48 h, or with 50 µM FCCP (carbonyl cyanide 4-(trifluoromethoxy) phenylhydrazone) for 60 min. FCCP was used to dissipate the mitochondrial membrane potential. After incubation time under 21% and 3% O_2_, the supernatant was removed and replaced with a solution of TMRE (1.2 µM) in Stain Buffer (BD Biosciences). After 30 min incubation at 37°C, the solution was removed and the wells were washed twice with Stain Buffer. Cells were detached with Accutase and collected into polypropylene tubes. Finally, cells were washed twice with Stain Buffer at room temperature and analyzed by flow cytometry. The median fluorescence intensity was calculated with the software FlowJo.

### Western blot analysis

After treatment, adherent and floating cells were harvested and washed twice with cold PBS. The cell pellets were lysed in RIPA buffer (150 mM NaCl, 0.1% Triton X-100, 0.5% sodium deoxycholate, 0.1% SDS, 20 mM Tris–HCl pH 7.5) supplemented with cOmplete™ EDTA-free protease inhibitor cocktail (Roche) for 30 min on ice. Lysates were centrifuged at 13,000 RPM for 20 min at 4 °C. Protein concentration was measured with Pierce™ BCA protein assay kit (Thermo Scientific). Samples of lysates were prepared in Laemmli buffer containing 100 mM DTT, boiled for 5 min at 95 °C and resolved in a 12% polyacrylamide gel. The following amount of protein was loaded per lane: 20 µg for Caspase-3, 25 µg for LC3 and p62 and 35 µg for BiP analyses. Transfer was performed onto nitrocellulose membrane via a semi-dry blotting system. Membranes were blocked in 5% nonfat milk powder in TBS-T buffer (Tris-base, NaCl and 0.1% Tween 20) for 1 h at room temperature. Following blocking, the membranes were cut in strips and strips were labeled overnight at 4 °C with the following antibodies against: Cleaved Caspase-3 (Cell Signaling, 1:1000; rabbit), LC3B (Cell Signaling, 1:1000; rabbit), SQSTM1/p62 (Cell Signaling, 1:1000; rabbit), BiP (Cell Signaling, 1:1000; rabbit), GAPDH (BioLegend, 1:1000; mouse), and GAPDH (Thermo Scientific, 1:1000; rabbit). After washing in TBS-T, membranes were incubated with HRP-conjugated secondary antibodies (anti-mouse IgG, Cell Signaling; or anti-rabbit IgG, Cell Signaling) for 1 h at room temperature, washed again and incubated with chemiluminescence reagent (Western Lightning Plus-ECL, Enhanced Chemiluminescence Substrate, PerkinElmer) for 1 min. Detection was performed with the chemiluminescent imaging system FusionCapt Advance FX7.

### Sphingolipid analysis

Sphingolipids were extracted from cell pellets using methyl-tert-butyl ether (MTBE)-based liquid–liquid extraction method (LLE). Cells were pelleted in Precellys tubes (Peqlab) to which steel balls were manually added. 1000 µl of MTBE/methanol (10:3, v/v), which served as an extraction solvent, were added to the tubes and then 250 µl of ice-cold 0.1% formic acid containing 5 µM tetrahydrolipstatin/3′-(aminocarbonyl) [1,1′-biphenyl]3-yl)-cyclohexylcarbamate and 10 µg/ml butyl hydroxytoluene as homogenization solvent. A 10 µl methanolic solution of internal standards: Sphingosine d17:1, Sphingosine-1P d17:1, Ceramide d18:1/17:0, Ceramide-1P d18:1/12:0, and Sphingomyelin d18:1/12:0 was then added to the tubes. The concentration of internal standards in the spike solution was set so as to result in a target concentration in the 100 µl final extracts of: 200 ng/ml for Sphingosine d17:1; 200 ng/ml for Sphingosine-1P d17:1; 500 ng/ml for Ceramide d18:1/17:0; 500 ng/ml for Ceramide-1P d18:1/12:0 and 100 ng/ml for Sphingomyelin d18:1/12:0. Samples were then homogenized for 20 s at 6000 rpm. Homogenates were transferred into 1.5 ml Eppendorf tubes, vortexed for 30 s at 4°C and centrifuged for 10 min at 13,000 rpm. Upper organic phase was recovered in new 1.5 ml tubes, evaporated to dryness under a gentle stream of nitrogen, reconstituted in 90 µl methanol and stored at -20°C till further analysis. Lower aqueous phase was used for protein content determination using the BCA assay. Lipids were analyzed by liquid chromatography/multiple reaction monitoring (LC/MRM) using QTRAP 5500 (AB Sciex) operating in positive/ negative ion mode switching. The ionization and detection parameters as well as LC solvents used for this analysis are as described in [31] and in [33] and are depicted in Additional file 1. Calibrants and MRM transitions for sphingomyelins, sphingosine and ceramide 1P species are as described in [34]. For the quantification of Ceramide d18:1/24:1 and Ceramide d18:1/16:0 in cell pellets, Ceramide d18:1/17:0 served as internal standard and Ceramide d18:1/16:0 was used as calibration standard. The MRM transitions for detection/quantification of ceramides were manually inferred by direct infusion analysis. The transitions and MRM parameters are listed in Additional file 2. For analysis an aliquot of 27 µl methanolic solution of lipid extract was mixed with 3 µl water and transferred in vials for liquid-chromatography/multiple reaction monitoring (LC/MRM) analysis. Lipid species were quantified using the Multiquant software version 3.0.3. The determined lipid concentrations were normalized to protein content of the cell pellets.

### Real-time quantitative PCR

Isolation of total RNA was performed using RNeasy Mini Kit (Qiagen) according to manufacturers’ instructions. After treatment, cells were collected, washed with 1xPBS and lysed in Buffer RLT (containing 1% ß-mercaptoethanol). All following steps were conducted as described in the manufacturers’ protocol. RNA concentration and quality were determined using a Nanodrop 2200 (ThermoFisher). Only samples showing a 260/280 nm ratio between 1.8 and 2.1 were selected for cDNA transcription, which was performed with the Omniscript RT Kit (Qiagen) and random hexamers (Life Technologies). Quantitative PCR (qPCR) analysis was done using TaqMan® primers and a StepOnePlus System (Applied Biosystems). Briefly, for each well of the 96-well qPCR plate (Sarstedt), 10 µl of TaqMan™ Universal PCR Master Mix (ThermoFisher) were mixed with 10 ng cDNA and 1 µl of the appropriate primer (ThermoFisher). All measurements were performed using three technical replicates. Relative quantification (RQ) of gene expression were determined using the 2^−ΔΔCt^ method. Primer IDs: DDIT3 (Hs00358796_g1), GAPDH (Hs02758991_g1); DEGS1 (Hs00186447_m1); SK1 (Hs00184211_m1); SK2 (Hs01016543_g1). Expression of GAPDH, used as a reference gene, was similar in untreated cells under both 21% and 3% oxygen conditions (Ct values of 20,743 and 20,749 respectively).

### Transmission Electron Microscopy (TEM)

NCH82 cells were cryo-fixed within a few milliseconds at a pressure of 2000 bar under liquid nitrogen using a high-pressure freezer Compact 1 (Wohlwend GmbH). Freeze-substitution was conducted using a Leica EM AFS 2 device (Leica Microsystems). Here, the substitution/staining medium (acetone p.a., 0.2% osmium tetroxide, 0.1% uranylacetate and 5% water) was pre-cooled to -90 °C before samples were added. Finally, the samples were embedded in EPON 812 and sectioned at room temperature using a diamond knife. Examination of the thin sections was conducted with a FEI Tecnai F20 transmission electron microscope (FEI) operated at an acceleration voltage of 200 kV. Conventional bright field images were acquired using a Gatan US1000 slow scan CCD camera (Gatan Inc.).

#### Statistical analysis

Data were expressed as the mean plus standard deviation (SD) of at least two independent experiments. Statistical analyses were performed using GraphPad Prism software (version 9.2.0). Mean comparison was performed using unpaired *t* test or ANOVA (one-way or two-way). Tukey and Šídák tests were used for multiple comparison analyses. The used statistical method is referred in the legend of the respective figure. Statistical significance was defined by an alpha of 0.05. * p ≤ 0.05; ** p ≤ 0.01; *** p ≤ 0.001; **** p ≤ 0.0001.

## Results

### TMZ synergizes with SKI-II to suppress glioblastoma cell growth under normoxia and hypoxia

**Fig. 1:**
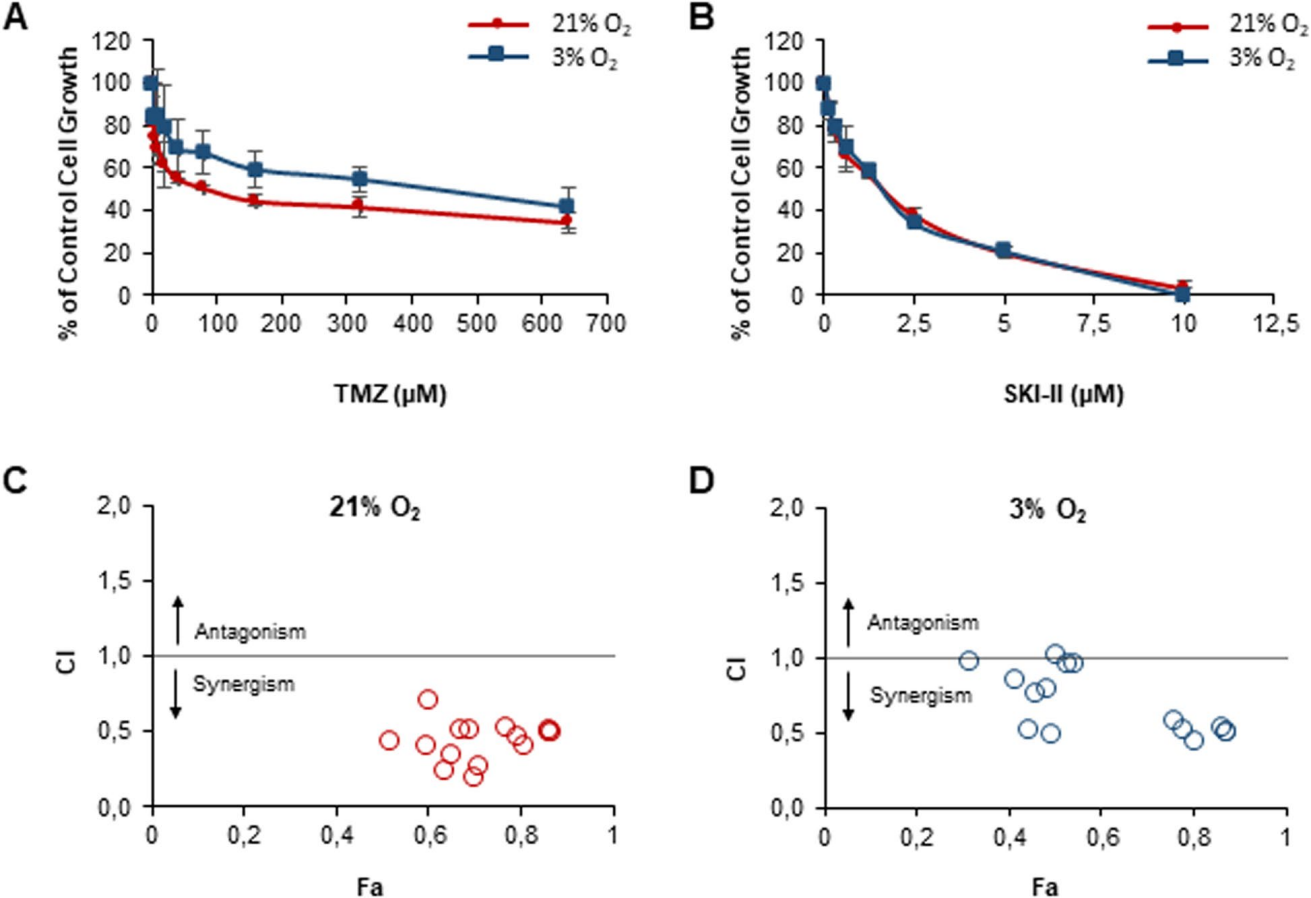
Hypoxia promotes resistance to temozolomide (TMZ) and affects its synergism with SKI-II. Dose–effect curves of (**A**) TMZ and (**B**) the sphingosine kinase inhibitor SKI-II on growth of NCH82 cells treated for 5 days. Data represent mean ± SD of *n* = 3. Combination index (CI) vs fraction affected (Fa) plots of 15 drug combinations of TMZ and SKI-II tested in NCH82 cells for 5 days at (**C**) 21% O_2_ and (D) 3% O_2_. CI = 1: additive effect. The sulforhodamine assay was used to determine the dose–effect curves and the CI. Data were obtained using the software CompuSyn from the mean of *n* = 3. Fa = 1 – (percentage of control cell growth/100)

The Chou-Talalay combination index method [25–27] was used to find the combination of TMZ and SKI-II with the strongest degree of synergism at the highest effect level towards the human glioblastoma NCH82 cells. First, dose–effect curves of each drug alone were determined under normoxia (21% O_2_) and hypoxia (3% O_2_) by exposing NCH82 cells to several twofold serial dilutions of TMZ and SKI-II (Fig. 1 A, B). Afterwards, the median effect dose (ED50 or D*m*), the *m* value (shape of the dose–effect curve) and the *r* value (conformity of the data to the mass-action law) were calculated using the software CompuSyn. These parameters are required to quantify synergism of drug combinations. The calculated ED50 for TMZ was 96 µM under normoxia and 428 µM under hypoxia, demonstrating a high resistance of the NCH82 cells to TMZ under low oxygen. On the contrary, SKI-II was as efficient under normoxia as under hypoxia in inhibiting cell growth (ED50 of approximately 1.3 μM in both oxygen conditions) (Additional file 3).

To evaluate the type and degree of interactions between SKI-II and TMZ, we next undertook to calculate the combination index (CI) for 15 combinations, where CI > 1 indicates antagonism, CI = 1 indicates an additive effect, and CI < 1 indicates synergism. SKI-II and TMZ were combined at a non-constant ratio and the concentrations of the 15 combinations were based on twofold serial dilutions above and below the ED50 of each drug (Additional file 4). TMZ concentrations were kept under clinically relevant ranges (3–100 µM; [21, 22]) and, therefore, not tested above 96 µM in combination.

All 15 combinations tested under normoxia had a CI value below 1 and are, accordingly, synergistic (Table 1). Under hypoxia some combinations became nearly additive (combinations A1 and C1-3) or presented a diminished degree of synergism (combinations B1-3) and their effect level was reduced. Interestingly, combinations with higher SKI-II concentrations, such as the combinations D1-3 (2 × ED50_SKI-II_) and E1-3 (4 × ED50_SKI-II_), presented a constant degree of synergism and similar effect level between oxygen conditions (Fig. 1 C, D; Table 1).

**Table 1:** Combination analysis of temozolomide (TMZ) and the sphingosine kinase inhibitor SKI-II

Drug Combo	TMZ (µM)	SKI-II (µM)	21% O_2_			3% O_2_		
			**Fa**	**CI**	**Degree**	**Fa**	**CI**	**Degree**
A1	24.0	0.33	0.52	0.43	+ + +	0.32	0.97	±
A2	48.0	0.33	0.64	0.24	+ + + +	0.44	0.51	+ + +
A3	96.0	0.33	0.70	0.18	+ + + +	0.49	0.48	+ + +
B1	24.0	0.66	0.60	0.39	+ + +	0.41	0.84	+ +
B2	48.0	0.66	0.65	0.33	+ + +	0.46	0.75	+ +
B3	96.0	0.66	0.71	0.26	+ + + +	0.48	0.79	+ +
C1	24.0	1.33	0.60	0.70	+ +	0.50	1.02	±
C2	48.0	1.33	0.67	0.51	+ + +	0.53	0.95	±
C3	96.0	1.33	0.69	0.50	+ + +	0.54	0.95	±
D1	24.0	2.66	0.76	0.52	+ + +	0.76	0.57	+ + +
D2	48.0	2.66	0.79	0.45	+ + +	0.78	0.51	+ + +
D3	96.0	2.66	0.81	0.40	+ + +	0.80	0.44	+ + +
E1	24.0	5.32	0.86	0.50	+ + +	0.87	0.50	+ + +
E2	48.0	5.32	0.87	0.49	+ + +	0.86	0.54	+ + +
E3	96.0	5.32	0.87	0.48	+ + +	0.87	0.51	+ + +

NCH82 cells were treated with 15 combinations of TMZ and SKI-II for 5 days under normoxia (21% O2) and hypoxia (3% O2). Combination Index (CI) was calculated from the CI equation algorithms using CompuSyn software. CI = 1, < 1 and > 1 indicates additive effect, synergism and antagonism, respectively. Fraction affected (Fa) and CI values of each drug combination were obtained from the average of three independent experiments. Graded symbols were attributed to the CI values to depict the degree of synergism of each combination, as suggested by Ting-Chau Chou (27): +  +  +  + , strong synergism; +  +  + , synergism; +  + , moderate synergism; ± , nearly additive

The synergistic drug combination consisting of 48 μM TMZ and 2.66 μM SKI-II (D2) was able to inhibit cell growth by almost 80% under normoxia and hypoxia (Table 1). The dose-reduction index (DRI), i.e. the measure of how many folds of dose-reduction for each drug at a given effect are allowed in synergistic combination, was obtained from the CompuSyn software (Additional file 5). The DRI of TMZ for this combination was of 84 under normoxia and 252 under hypoxia. Thus, the synergistic (TMZ + SKI-II) combination was able to reduce the needed concentration of TMZ by 252-fold under hypoxia. The DRI of SKI-II was as well favorable (DRI > 1) and comparable under both oxygen conditions (2.2 in normoxia and 1.9 in hypoxia). Combinations with a higher degree of synergism at a higher effect level are the most relevant for cancer therapy [28]. For this reason, and in order to combine a clinically relevant dose of TMZ with the lowest concentration of SKI-II, we decided to use the D2 (TMZ + SKI-II) combination for further studies.

### The combination of TMZ and SKI-II potentiates glioblastoma cell death under normoxia and hypoxia

**Fig. 2:**
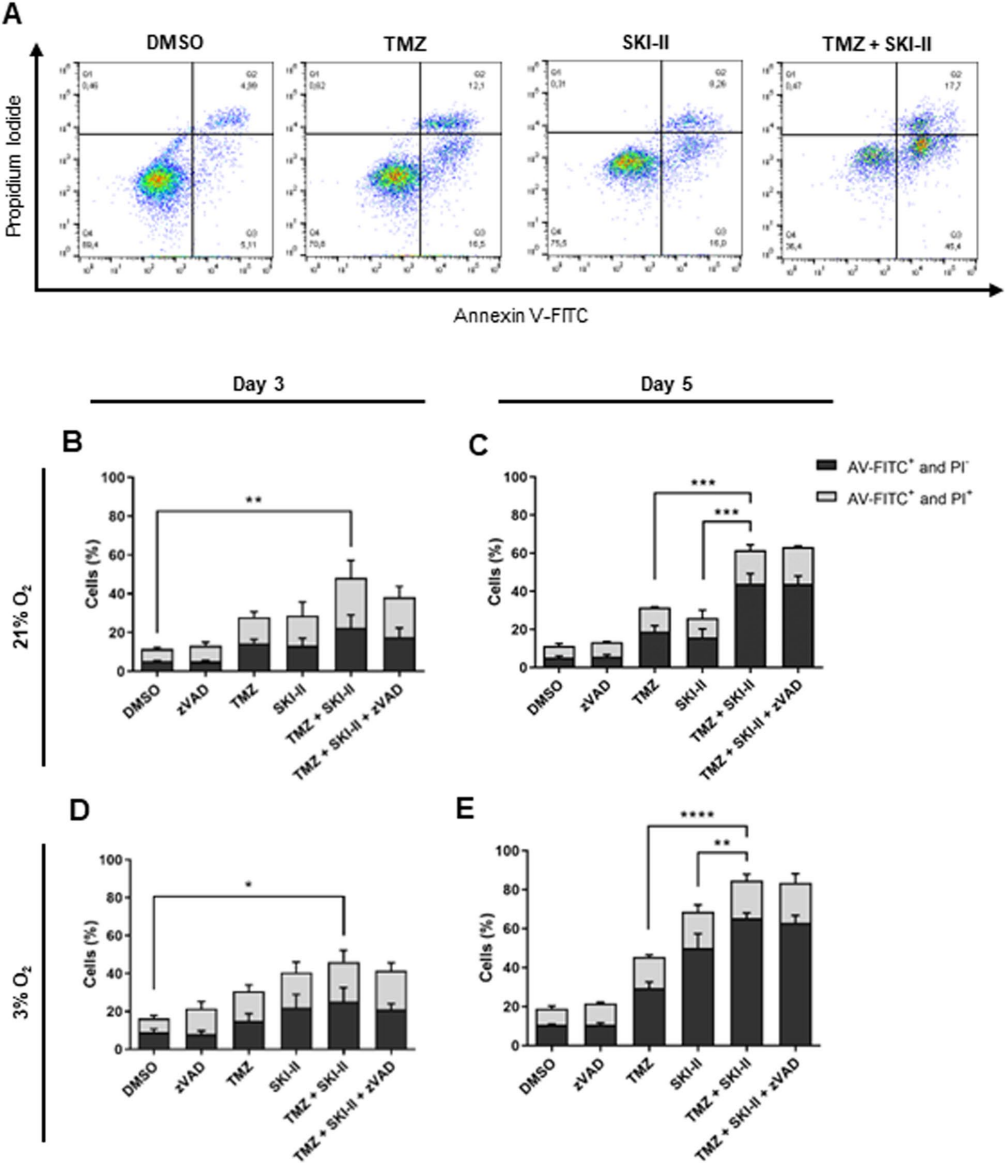
The combination of temozolomide (TMZ) and SKI-II potentiates death in GB cells. NCH82 cells were analyzed by flow cytometry after treatment with vehicle control (DMSO), 48 µM TMZ, 2.66 µM SKI-II, and the combination (TMZ + SKI-II) in the presence and absence of 20 µM zVAD-fmk (zVAD) for 3 and 5 days, under normoxia (21% O_2_) and hypoxia (3% O_2_). **A** Applied quadrant in propidium iodide- (PI) and AnnexinV (AV)-FITC-stained NCH82 cells. **B**-**E** Quantification of PI- and AV-FITC-labeled NCH82 cells. Data represent mean + SD of n = 3. Flow cytometry data analysis was performed using the software FlowJo. One-way ANOVA followed by Tukey’s multiple comparisons test was performed for statistical analysis

To quantify cell death induced by (TMZ + SKI-II) combination and each drug alone, NCH82 cells were treated for 3 and 5 days under normoxia and hypoxia. After treatment, cells were stained with annexin-V-FITC (AV-FITC) and propidium iodide (PI) and, subsequently, analyzed by flow cytometry (Fig. 2 A). Under normoxia, single treatment with TMZ or SKI-II induced similar levels of cell death (approximately 30% of AV-FITC^+^/PI^−^ and AV-FITC^+^/PI^+^ cells) after 3 and 5 days of treatment, whereas the level of cell death induced by the combination was significantly enhanced by about 30% after 5 days (Fig. 2 B, C). Under hypoxia, the combination and single treatments were similarly cytotoxic after 3 days, and this cytotoxicity increased after 5 days. The combination significantly potentiated cell death in comparison with TMZ alone after 5 days of treatment (Fig. 2 D, E).

Altogether, the present data show that treatment with the (TMZ + SKI-II) combination was, both under normoxia and hypoxia, more efficient in killing the glioblastoma NCH82 cells than single TMZ or SKI-II treatment, and this effect was time-dependent.

### The combination induces cell death without caspase-3 activation or mitochondrial membrane potential disruption

**Fig. 3:**
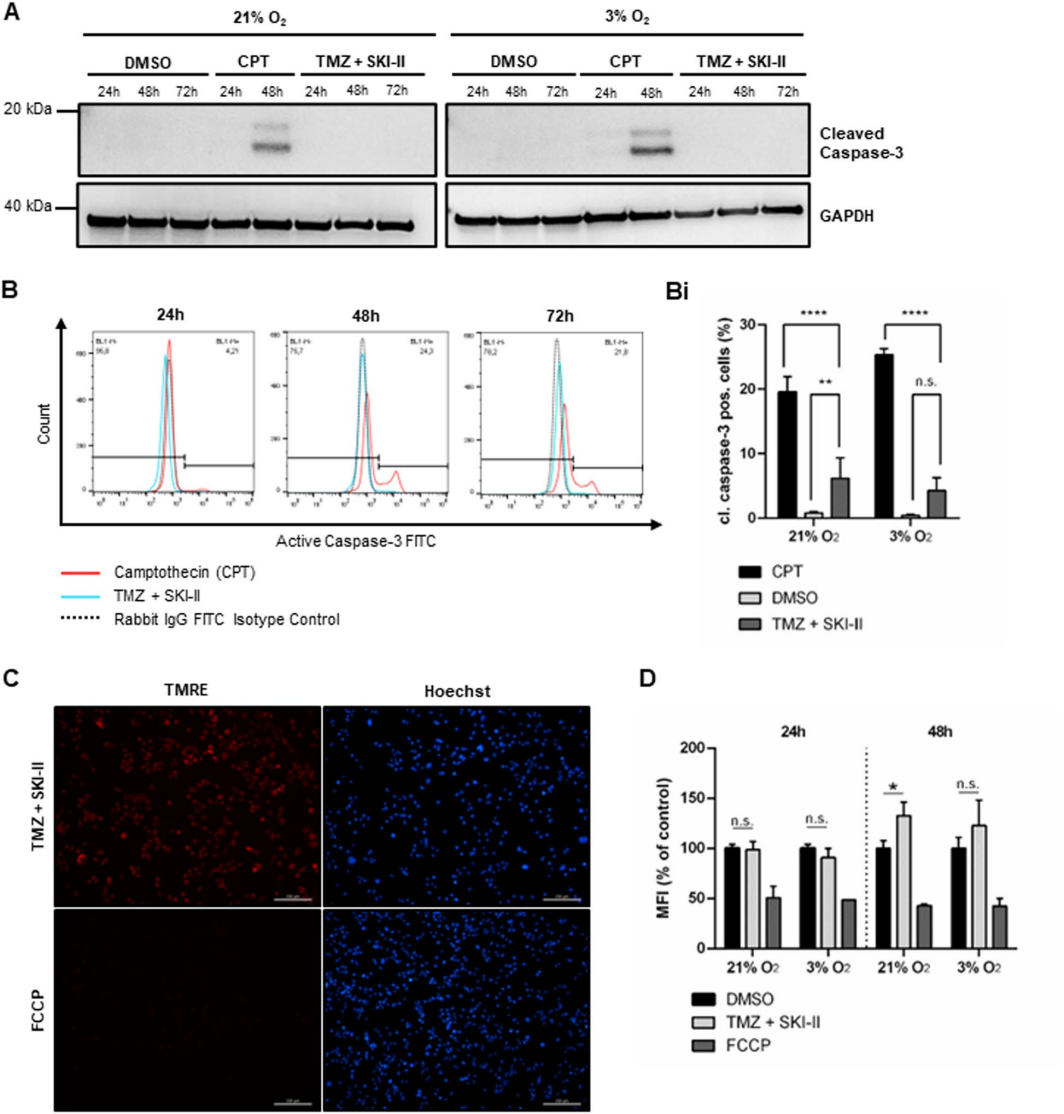
Combination-induced cell death occurs without caspase-3 activation and disruption of the mitochondrial membrane potential. **A** Western blot analysis of cleaved caspase-3 in NCH82 cells treated with the vehicle control (DMSO), 15 µM Camptothecin (CPT) and the combination of 48 µM TMZ and 2.66 µM SKI-II (TMZ + SKI-II). Full-length blots are presented in Additional file 6. (B, Bi) Flow cytometric quantification of active caspase-3 FITC-positive NCH82 cells treated with the vehicle control (DMSO), 1 µM Camptothecin (CPT) and (TMZ + SKI-II) for 72 h under normoxia (21% O_2_) and hypoxia (3% O_2_). **B** Representative plots of NCH82 cells treated under hypoxia. (Bi) Analysis and quantification of the data was performed with the software FlowJo. Data are expressed as the mean (± SD) of the percent of total single cells; *n* = 3. One-way ANOVA followed by Tukey’s multiple comparisons test was performed for statistical analysis. Analysis of the mitochondrial membrane potential by TMRE labelling in NCH82 treated cells via (**C**) live cell fluorescence microscopy after 48 h at 21% O_2_ and (**D**) flow cytometry after 24- to 48 h under normoxia and hypoxia. Treatment with the depolarization control FCCP (50 µM) for 60 min. The median fluorescence intensity (MFI) of TMRE was calculated with FlowJo. Data represent mean (± SD) of *n* = 3. Two-way ANOVA followed by Tukey’s multiple comparisons test was performed for statistical analysis. Scale bar = 200 µm

Apoptosis can occur via the extrinsic and intrinsic signaling pathways. Both pathways culminate in the activation of effector caspases, such as caspase-3 [35]. Addition of the pan-caspase inhibitor Z-VAD-FMK (zVAD) did not rescue the NCH82 cells from death induced by the combination (Fig. 2 B-E). To confirm this observation, we analyzed the effect of the combination on caspase-3 activation over 72 h of treatment under hypoxia and normoxia. As shown in Fig. 3, cleaved caspase-3 was not detected in cells treated with the combination after 48 h (Fig. 3 A, B, Bi; Additional file 6) and was expressed in approximately 5% or less of the cells analyzed after 72 h of treatment. This indicates that the majority of cells dies via a caspase-independent cell death pathway (Fig. 3 B, Bi). Note that NCH82 cells do activate caspase-3, as shown by the detection of the cleaved caspase-3 after treatment with camptothecin (CPT), a well-known apoptosis inducer (Fig. 3 A, B, Bi; Additional file 6). Altogether, these data suggest that the combination induces a mode of cell death independent of caspase activation.

During apoptosis, mitochondrial outer membrane permeabilization leads to a loss of mitochondrial membrane potential (MMP) – an event occurring before caspase activation. To analyze whether the (TMZ + SKI-II) combination causes alterations in the MMP, we stained treated NCH82 cells with tetramethylrhodamine ethyl ester (TMRE), a fluorescent dye only able to bind mitochondria with an intact MMP. Fluorescence microscopy showed that treatment with (TMZ + SKI-II) did not cause the loss of TMRE fluorescence which was observed in the FCCP-treated cells—a positive control for the dissipation of the MMP (Fig. 3 C). Quantification via flow cytometry confirmed that FCCP treatment caused a significant decrease of approximately 50% of the median fluorescence intensity (MFI) of TMRE. The MFI of cells treated with (TMZ + SKI-II) did not significantly differ from the MFI of control cells (DMSO condition) at 24 h and 48 h, except for an increase observed at 48 h under normoxia (Fig. 3 D). This increased fluorescence, that could be interpreted as an hyperpolarization, is most likely to result from an increase in cell death at late time points leading to an increase in the dye-to-living cells ratio, as described in [36]. These results indicate that the combination does not cause mitochondrial depolarization under both oxygen conditions, and therefore, mitochondria do not seem to have a decisive role in (TMZ + SKI-II)-induced cell death.

### The combination of TMZ and SKI-II does not affect autophagy in normoxia and hypoxia

We and others previously reported that TMZ and SKI-II are able to induce autophagy [16, 18, 37]. Therefore, autophagy could have an important role in, or at least, accompany (TMZ + SKI-II)-induced cell death. To monitor autophagic flux, we analyzed LC3-II and p62 levels in the presence and absence of bafilomycin A1 (BA1). BA1 is a specific inhibitor of the vacuolar type H^+^-ATPase and inhibits the acidification of organelles containing this enzyme (lysosomes and endosomes). Thus, it blocks the process of autophagy at a late stage. The use of BA1 supports the interpretation of LC3 immunoblotting, since an increase in LC3-II levels may represent an increase in autophagy or a block in the autophagy flux [38].

NCH82 cells were treated with the combination and DMSO control for 24 to 72 h under normoxia and hypoxia. Under normoxia, p62 and LC3-II protein levels remained similar between both conditions over time (Additional file 7 A, Ai, Aii; Additional file 8). The autophagic flux in (TMZ + SKI-II)-treated cells decreased from 24 to 48 h, but there were no differences in comparison with control cells (Additional file 7 Aiii). Under hypoxia, p62 protein levels were not significantly affected by the combination whereas LC3-II levels at 72 h were elevated in (TMZ + SKI-II)-treated cells in comparison with the DMSO condition (Additional file 7 B, Bi, Bii; Additional file 8). Indeed, treatment with the combination tended to decrease autophagic flux over time—however, this lacks statistical significance (Additional file 7 Biii). This trend was not observed in DMSO-treated cells, but as in normoxia, there were no statistically significant differences in the autophagic flux in comparison with the (TMZ + SKI-II)-treated cells. These results suggest that TMZ combined with SKI-II at low-doses does not significantly affect autophagic flux and, therefore, autophagy does not seem to be an essential process in cell death.

### SKI-II alone and in combination increases cytoplasmic vacuolization

**Fig. 4:**
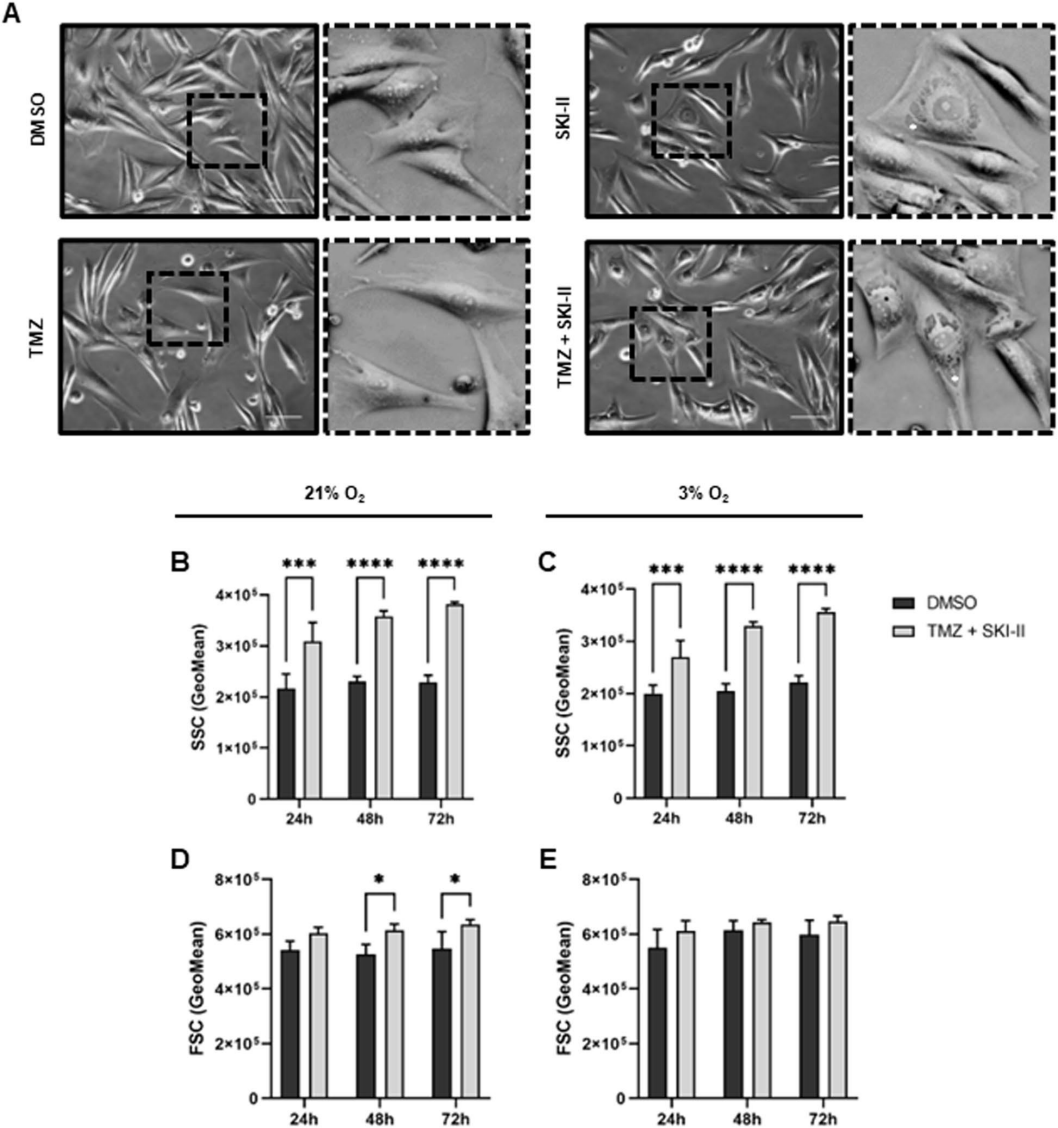
SKI-II alone and in combination with temozolomide (TMZ) increases cytoplasmic vacuolization in glioblastoma cells. **A** Light microscopy of NCH82 cells treated for 72 h at 3% O_2_ with the vehicle control (DMSO), 48 µM TMZ, 2.66 µM SKI-II, and the combination (TMZ + SKI-II). **B**-**E** NCH82 cells were treated accordingly for 24- to 72 h under normoxia (21% O_2_) and hypoxia (3% O_2_) and further analyzed by flow cytometry. The light scattering parameters SSC (side scatter) and FSC (forward scatter) were quantified with FlowJo. Data represent mean (+ SD) of n = 3. Scale bar: 100 µm. Two-way ANOVA followed by Šídák's multiple comparisons test was performed for statistical analysis

Microscopy observations indicated that SKI-II alone or in combination with TMZ induces cytoplasmic vacuolization in NCH82 cells (Fig. 4 A). In order to try and quantify this increase, we used flow cytometry to take advantage of the side scatter (SSC) parameter that is a measure of the cell granularity. The SSC of (TMZ + SKI-II)-treated cells was significantly increased in comparison to that of control cells (DMSO vehicle) under normoxia and hypoxia. This increase was evident after 24 h and aggravated over time. It should be noted that the formation of cytoplasmic vacuoles was observed at the microscope as early as 8 h after treatment (data not shown). SSC values observed in combination-treated cells were similar in both oxygen conditions (Fig. 4 B, C). The size of the cells, measured via the forward scatter (FSC, Fig. 4 D, E), was slightly increased after (TMZ + SKII) treatment under normoxia at later time points. These differences were not observed in hypoxia. Altogether these results indicate cytoplasmic vacuolization to be an early effect of sphingosine kinase inhibitor alone and in combination with TMZ.

### The combination of TMZ and SKI-II induces ER stress and the Unfolded Protein Response

**Fig. 5:**
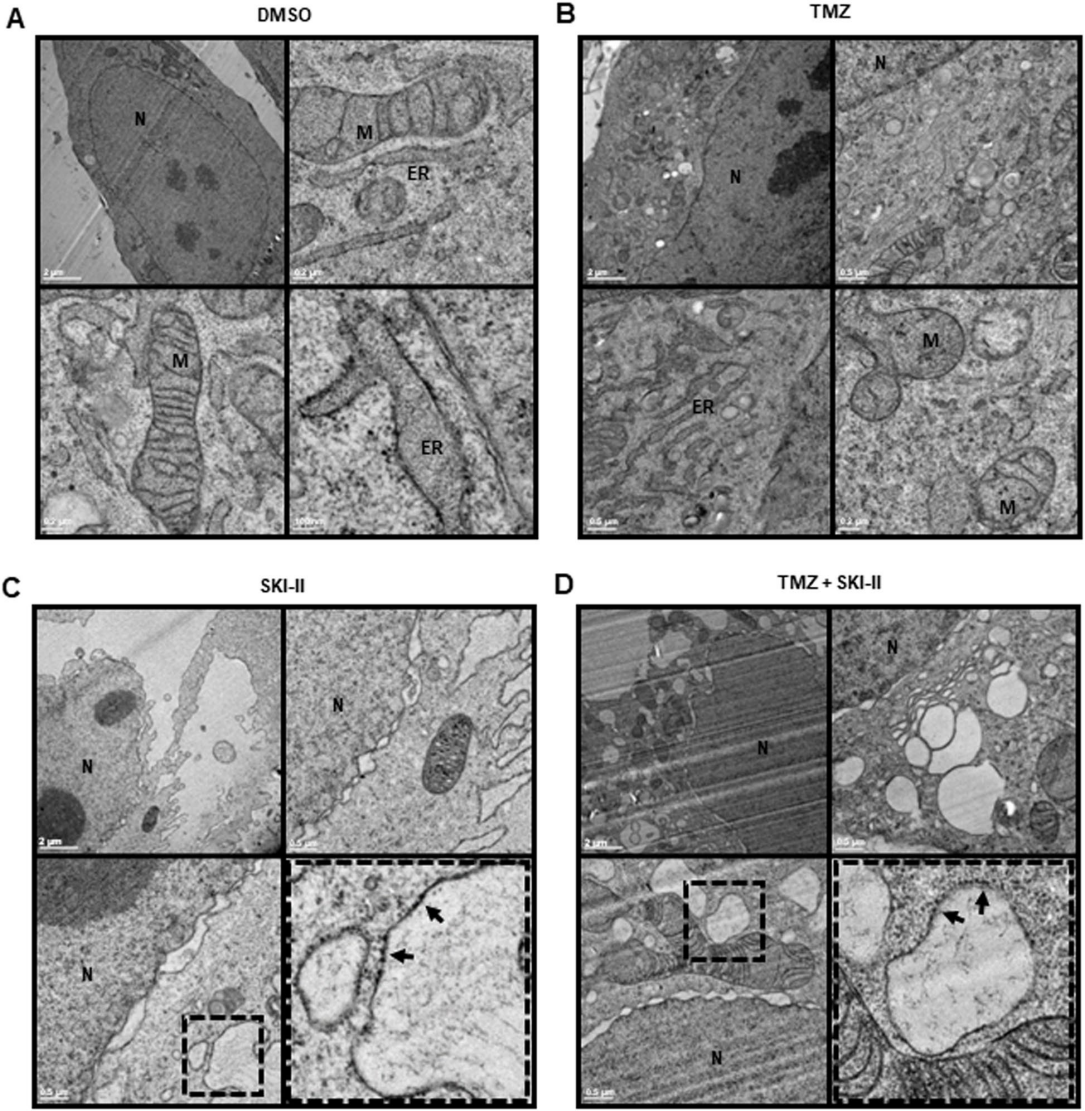
SKI-II alone and in combination with temozolomide (TMZ) induces dilation and fragmentation of the ER. NCH82 cells were treated at 3% O_2_ with (**A**) the vehicle control (DMSO), (**B**) 48 µM TMZ, (**C**) 2.66 µM SKI-II, and (**D**) the combination (TMZ + SKI-II) for 48 h and further analyzed via transmission electron microscopy. Dotted squares are regions of interest. N – nucleus; M – mitochondria; ER – endoplasmic reticulum. Black arrows show ribosomes

Based on our previous observations and reported effects of SKI-II on ER dilation and expansion [18], we further examined the cytoplasmic vacuolization in NCH82 treated cells by transmission electron microscopy (TEM). In control cells (DMSO) and in TMZ-treated cells, the ER presented a normal tubular morphology (Fig. 5 A, B). In cells treated with SKI-II alone and in combination with TMZ, we observed an enlargement but also fragmentation of the rough ER (vesicles with ribosomes) (Fig. 5 C, D). Thus, the cytoplasmic vacuolization observed by light microscopy could represent an expanded and dilated ER. Dilation of the nuclear envelope was also observed in cells treated with SKI-II and with the combination. This is not surprising, since the outer nuclear membrane is directly continuous with the rough ER. These ultrastructural changes were observed under both oxygen conditions (21% O_2_ not shown). Untreated NCH82 cells commonly presented disarrangement and distortion of mitochondrial cristae. Such abnormalities have been previously described in GB [39].

**Fig. 6:**
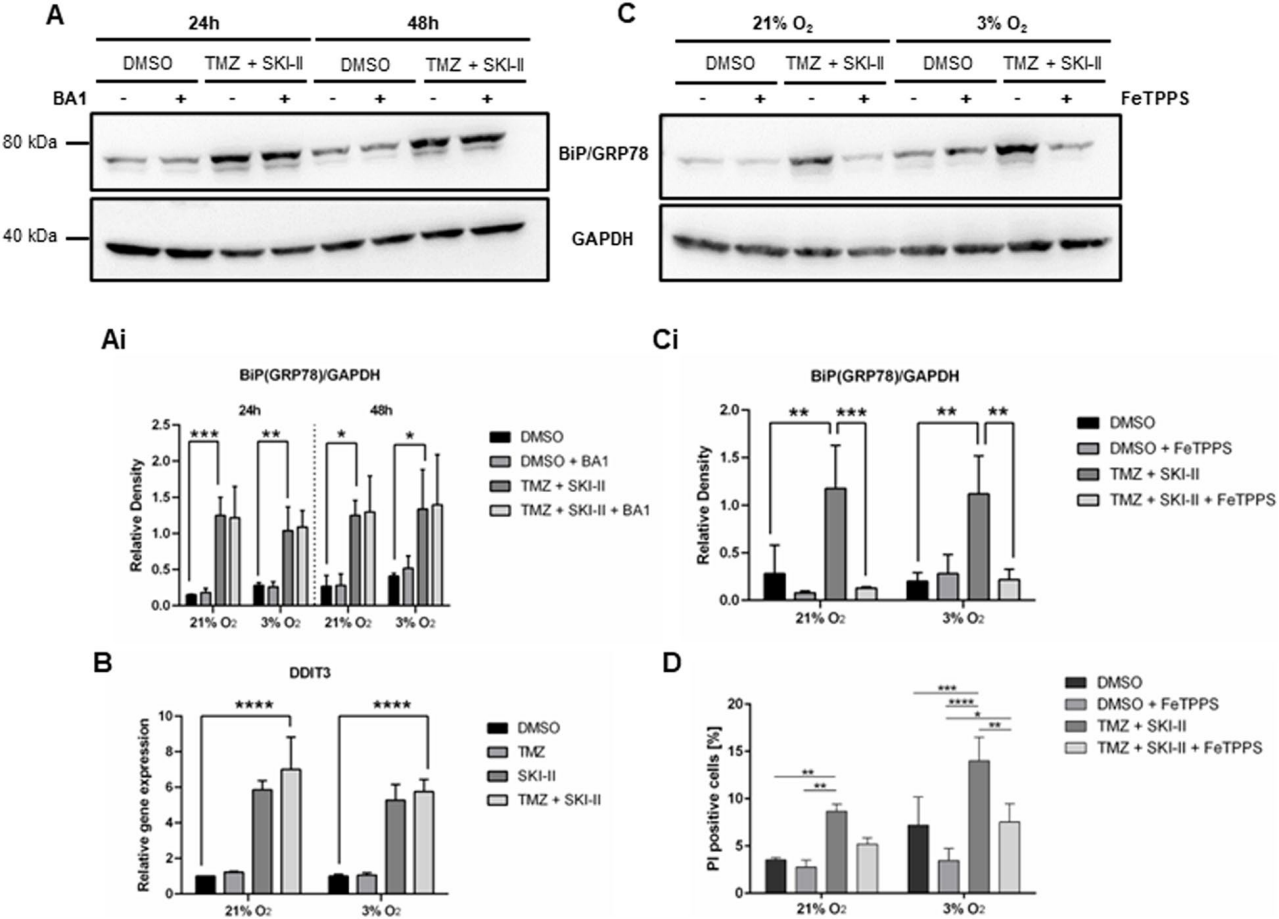
A peroxynitrite decomposition catalyst rescues the combination effects on ER stress, cytoplasmic vacuolization and viability. (A, Ai) Western Blot analysis of BiP/GRP78 in NCH82 cells treated with the vehicle control (DMSO) and 48 µM TMZ in combination with 2.66 µM SKI-II (TMZ + SKI-II) in absence or presence of Bafilomycin A (BA1), for 24- to 48 h under normoxia (21% O_2_) and hypoxia (3% O_2_). GAPDH was used as a loading control. **A** Representative blot of cells treated under 3% O_2_. Full-length blots are presented in Additional file 9. (Ai) BiP protein expression relative to GAPDH was quantified with ImageJ. **B** Gene expression analysis of DDIT3 in NCH82 cells treated accordingly for 24 h under normoxia and hypoxia. Data represent mean (± SD) of *n* = 3. Two-way ANOVA followed by Tukey’s multiple comparisons test was performed for statistical analysis. **C** Western blot analysis of BiP/GRP78 in NCH82 cells treated accordingly for 24 h under normoxia (21% O_2_) and hypoxia (3% O_2_). FeTPPS was used at a final concentration of 100 µM. A representative blot is shown. Full-length blots are presented in Additional file 10. (Ci) BiP/GRP78 protein expression level was quantified and normalized to GAPDH with ImageJ. **D** Viability of cells treated accordingly for 72 h was determined via the PrestoBlueTM assay. Data were normalized to the DMSO condition. Data represent mean (± SD) of *n* = 3. Two-way ANOVA followed by Tukey’s multiple comparisons test was performed for statistical analysis

These morphological changes suggest that, similar to cells treated with a higher dose combination [18], cells treated with the (TMZ + SKI-II) combination used in the present study underwent ER stress. To confirm this, we analyzed the protein levels of the ER chaperone BiP (Binding immunoglobulin Protein) also known as GRP78 (glucose regulatory protein 78), and the expression of Unfolded Protein Response (UPR) target gene *DDIT3* coding for CHOP (C/EBP Homologous Protein). Under stress conditions, BiP dissociates from ER transmembrane proteins resulting in the activation of the UPR. Subsequently, BiP expression is upregulated to support the re-establishment of ER homeostasis. Prolonged UPR activation leads to the induction of the pro-apoptotic transcription factor CHOP. Treatment of NCH82 cells up to 48 h with the combination resulted in upregulation of BiP expression levels. Co-treatment with Bafilomycin A1 did not affect BiP expression, suggesting that autophagy does not have a role in the mitigation of ER stress (Fig. 6 A-Ai; Additional file 9). In contrast to TMZ treatment, SKI-II alone or in combination elevated *DDIT3* expression levels by about sixfold, showing that ER stress levels are increased due to SKI-II treatment solely (Fig. 6 B). Hypoxia does not seem to aggravate ER stress, since BiP and *DDIT3* levels were similar in both oxygen conditions. Altogether, these data indicate that SKI-II alone or in combination with TMZ induces ER stress in NCH82 cells.

### ER stress and cytotoxicity induced by the combination of TMZ and SKI-II are peroxynitrite-dependent

ER stress and the generation of reactive oxygen and nitrogen species are linked events [40]. Peroxynitrite, a short-lived oxidant species produced by the reaction of nitric oxide with superoxide radicals, can induce ER stress via depletion of ER-Ca^2+^ [41]. We previously reported that FeTPPS, a decomposition catalyst of peroxynitrite, protects NCH82 cells from ER stress and from death induced by the high-dose (TMZ + SKI-II) combination [18]. To determine the contribution of peroxynitrite to the ER stress and viability loss induced by the lower doses of the (TMZ + SKI-II) combination, we co-treated NCH82 cells with 100 µM FeTPPS. Mitigation of peroxynitrite significantly decreased BiP protein levels in (TMZ + SKI-II)-treated cells (Fig. 6 C-Ci; Additional file 10) as well as the number of dead cells induced by the treatment, under both normoxia and hypoxia (Fig. 6 D). Altogether, these results suggest that oxidative stress has an important role in ER stress and cell death induced by the combination.

### SKI-II alone and in combination reduces the levels of ceramide and its metabolites

**Fig. 7:**
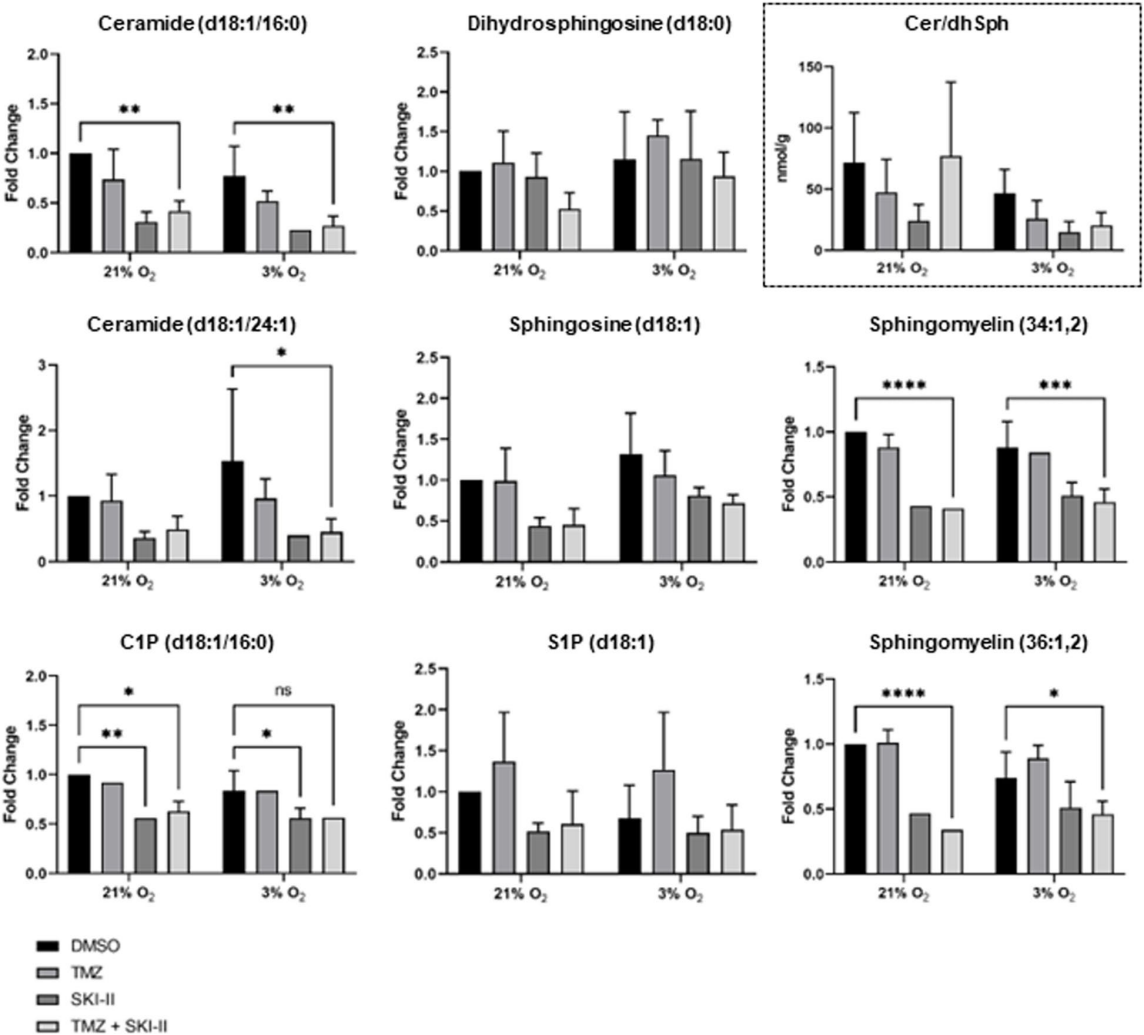
SKI-II alone and combined with temozolomide (TMZ) reduces the levels of ceramide, and ceramide-derived metabolites. NCH82 cells were treated for 48 h with the vehicle control (DMSO), 48 µM TMZ, 2.66 µM SKI-II and the combination (TMZ + SKI-II) under normoxia (21% O_2_) and hypoxia (3% O_2_). Sphingolipids were quantified via liquid chromatography mass spectrometry. Data are normalized as fold change ratio of treatment to DMSO control values at 21% O2 and represent mean (+ SD) of *n* = 3 (Ceramide-1-Phosphate, *n* = 2). The framed panel Cer/dhSph shows the ratio of Cer (d18:1/16:0) to dihydrosphingosine and indicates that SKI-II affects the flux of the de novo synthesis of ceramide. The ratio was determined from the mean (+ SD) of *n* = 3. Two-way ANOVA followed by Tukey’s multiple comparisons test was performed for statistical analysis. Amount of sphingolipid present in DMSO control cells at 21% O_2_ (mean ± SD): C24-ceramide, 4791.2 ± 1790.9 nmol/g; C16-ceramide, 18,128.9 ± 8185.5 nmol/g; C16-ceramide-1-phosphate, 16,431 ± 221.3 nmol/g; sphingosine-1-phosphate, 81,925.5 ± 33,204.2 nmol/g; sphingosine, 656.6 ± 96.6 nmol/g; dihydrosphingosine, 283.1 ± 85.3 nmol/g; sphingomyelin 36:1,2, 975.1 ± 489.9 nmol/g; sphingomyelin 34:1,2, 35,030.9 ± 8629.9 nmol/g

**Fig. 8:**
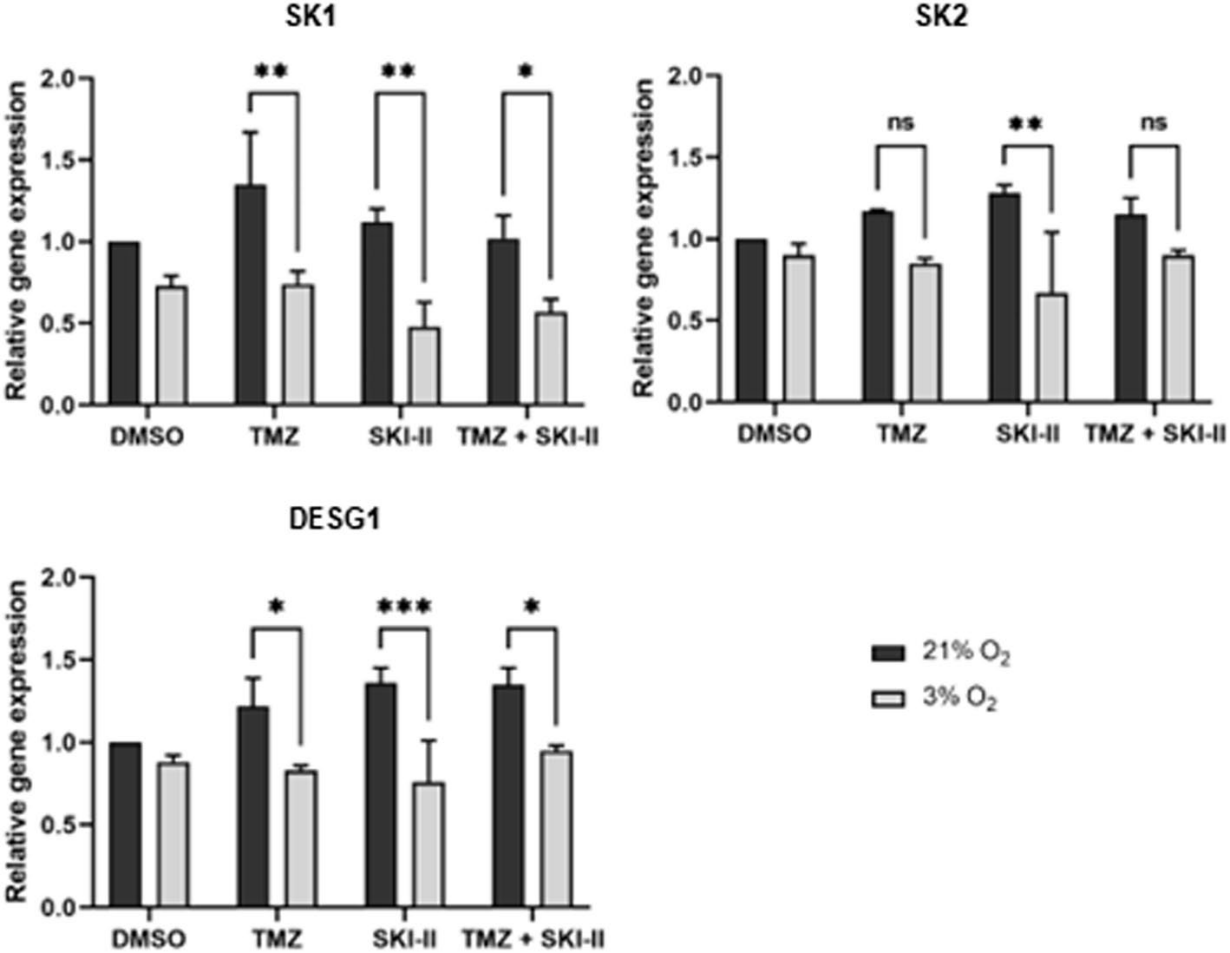
Reduced expression of *SK1*, *SK2* and *DESG1* genes after treatment under hypoxic conditions. NCH82 cells treated with the vehicle control (DMSO), 48 µM TMZ, 2.66 µM SKI-II and the combination (TMZ + SKI-II) for 24 h under normoxia (21% O_2_) and hypoxia (3% O_2_) were analyzed for the expression of *SK1* (sphingosine kinase 1); *SK2* (sphingosine kinase 2); and *DESG1* (dihydroceramide desaturase 1) by RT-qPCR. Data represent mean (± SD) of *n* = 3. Two-way ANOVA followed by Tukey’s multiple comparisons test was performed for statistical analysis

We next assessed the effects of the treatments on the sphingolipid metabolism by measuring the concentration of relevant metabolites in NCH82 cells. Treatment for 24 h (not shown) and 48 h (Fig. 7) with SKI-II alone and in combination led to a significant decrease in ceramide (d18:1/16:0) in both oxygen conditions; the same trend was observed for ceramide (d18:1/24:1) with a significant decrease under hypoxia. The level of the ceramide precursor dihydrosphingosine (dihydroceramide was not detectable) was not significantly affected by the treatments. The ceramide/dihydrosphingosine ratio was reduced by SKI-II in both oxygen conditions. It was not affected by the combination under normoxia, but was reduced under hypoxia. Sphingomyelin, ceramide-1-phosphate (C1P) and, to a lesser extent, sphingosine, which are all direct metabolites of ceramide, showed a profile similar to that of ceramide under normoxia and hypoxia. Similar to sphingosine, S1P levels were decreased by SKI-II alone and in combination at 21% O_2_ (although not significantly), and remained unchanged at 3% O_2_. TMZ did not induce major alterations in the sphingolipid levels. Furthermore, none of the treatments significantly affected the basal level of expression of the genes coding for SK1, SK2, and DES1 in cells treated under either oxygen concentration (Fig. 8). Altogether, these data suggest that changes in sphingolipid levels after treatment with SKI-II alone and in combination were primarily the consequences of changes in ceramide levels.

### The combination impairs the self-renewal of resistant glioblastoma stem cells

**Fig. 9:**
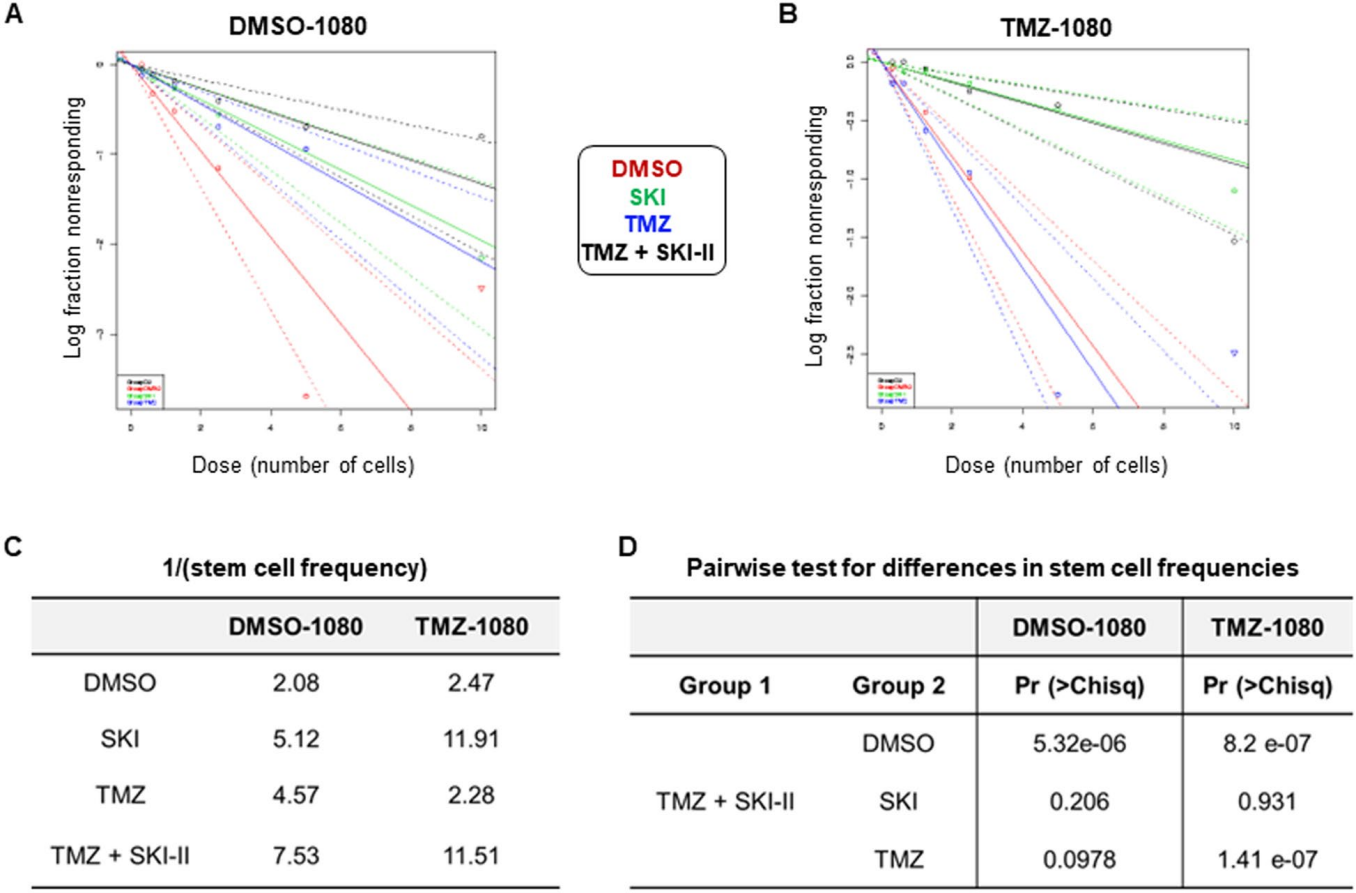
The combination affects the self-renewal capacity of TMZ-resistant glioblastoma stem-like cells (GSCs). Stem cell frequency was determined by extreme limiting dilution assay (ELDA) in DMSO-selected 1080 GSCs and TMZ-selected 1080 GSCs. Cells were treated with the DMSO control (red line), 48 µM TMZ (blue line), 2.66 µM SKI-II (green line) and the combination (TMZ + SKI-II) (black line) for 4 weeks under normoxia (21% O_2_; *n* = 3). **A**, **B** Log-fraction plots, where the y axis “log fraction nonresponding” indicates frequency of cells incapable of forming clonal spheres and the x axis “dose (number of cells)” indicates number of cells per ml. The slope of the line is the log-active cell fraction, and dotted lines give the 95% confidence interval. Note that the panels in A and B have slightly different Y-axis scales. **C** Estimated stem cell frequency, 1/(stem cell frequency). **D** Pairwise test for differences in stem cell frequencies between groups. Data analysis was performed via the ELDA webtool (see Additional file 11 for complete ELDA results)

We performed extreme limiting dilution analysis (ELDA) to determine the effect of the (TMZ + SKI-II) combination on the self-renewal capacity of glioblastoma stem cells (GSCs) under hypoxia and normoxia. Limiting dilution assays allow the quantification of the frequency of biologically active cells in a population. Two GSC lines were used: the control line DMSO-1080 and the line TMZ-1080, selected for its resistance to 100 µM TMZ. Whereas single treatments and the combination decreased the stem cell frequency of the control DMSO-1080 cells under normoxia (Fig. 9), only SKI-II and the combination affected this frequency under hypoxia (Additional file 11). The self-renewal capacity of TMZ-1080 cells was not affected by TMZ, as expected. Treatment with SKI-II and the combination on the contrary impaired this capacity, particularly under hypoxia. The stem cell frequency decreased almost fourfold in comparison with SKI-II alone, yet it remained similar under normoxia (Fig. 9; Additional file 11).

**Fig. 10:**
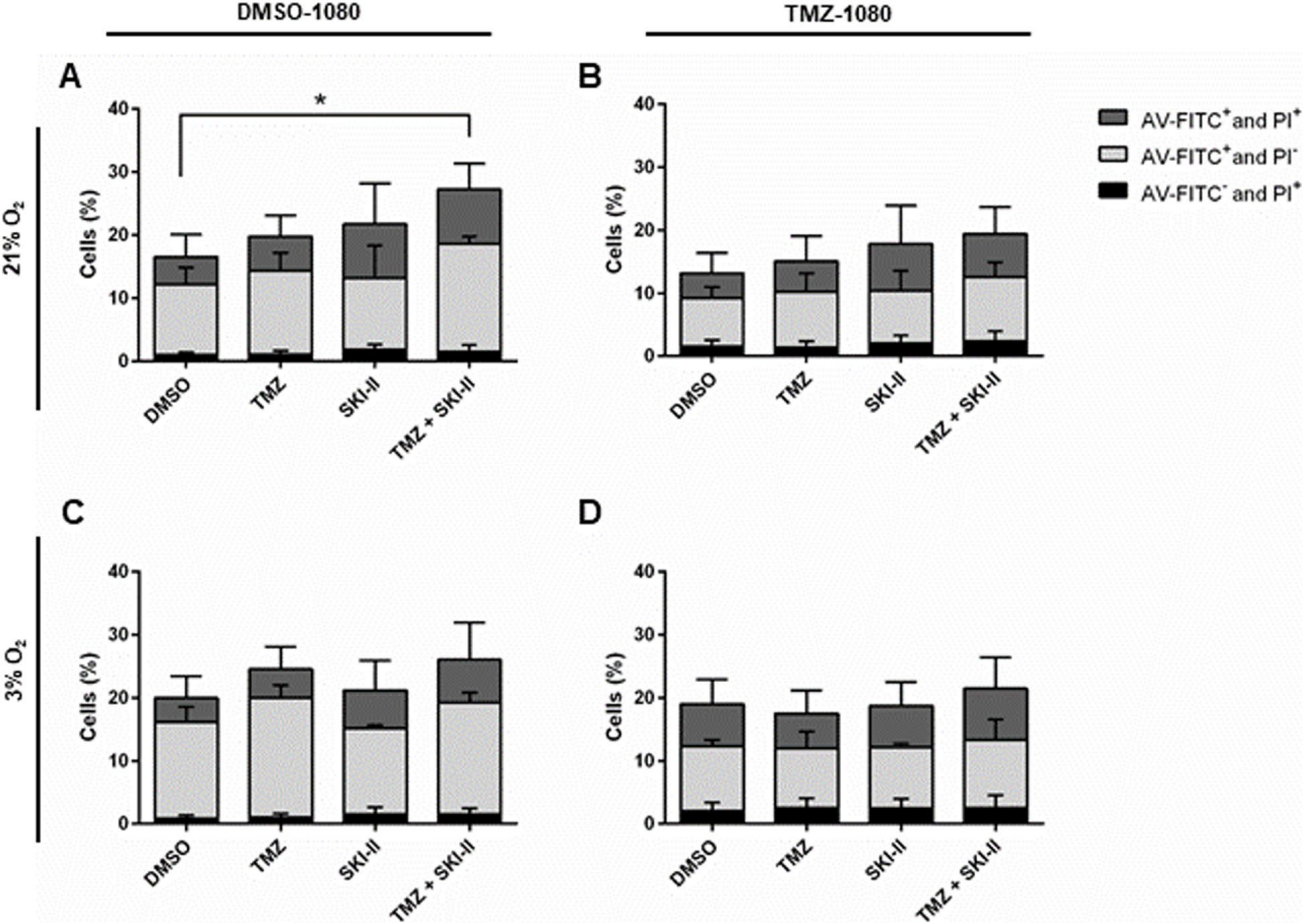
The combination exerts a very weak cytotoxic effect on GSC cells. The 1080-DMSO and 1080-TMZ cells were analyzed by flow cytometry after treatment with vehicle control (DMSO), 48 µM TMZ, 2.66 µM SKI-II, and the combination (TMZ + SKI-II) for 5 days under normoxia (21% O_2_) and hypoxia (3% O_2_). Quantification of PI- and AV-FITC-labeled GSC cells was performed using the software FlowJo. Data are presented as percent of total cells; *n* = 3

We next examined the cytotoxic potential of the single and combined treatments towards the DMSO-1080 and TMZ-1080 cells. As shown in Fig. 10, a significant cell death induction could not be detected in either GSC line after 5 days of TMZ treatment. A slight though not significant increase in cell death was detectable after treatment with SKI-II alone under normoxia, but not under hypoxia. The combinatorial treatment led to a significant increase in cell death of DMSO-1080 cells at 21% O_2_, but not in any of the other conditions. We did not detect significant differences in the distribution of early apoptotic (AnnV + /PI-), late apoptotic (AnnV + /PI +) or necrotic (AnnV-/PI +) cells between the conditions. Immunofluorescence analysis of the GSC lines indicated the presence of cells dying via apoptosis as evidenced by morphological features and activation of caspase 3 (Additional file 12). This however represented a low percentage of cells. Altogether these data indicate that SKI-II alone and in combination efficiently reduce the self-renewal capacity of the two GSC lines without exhibiting significant cytotoxic effects.

### SKI-II alone or in combination impairs invasion of mesenchymal MGMT-positive GB cells

**Fig. 11:**
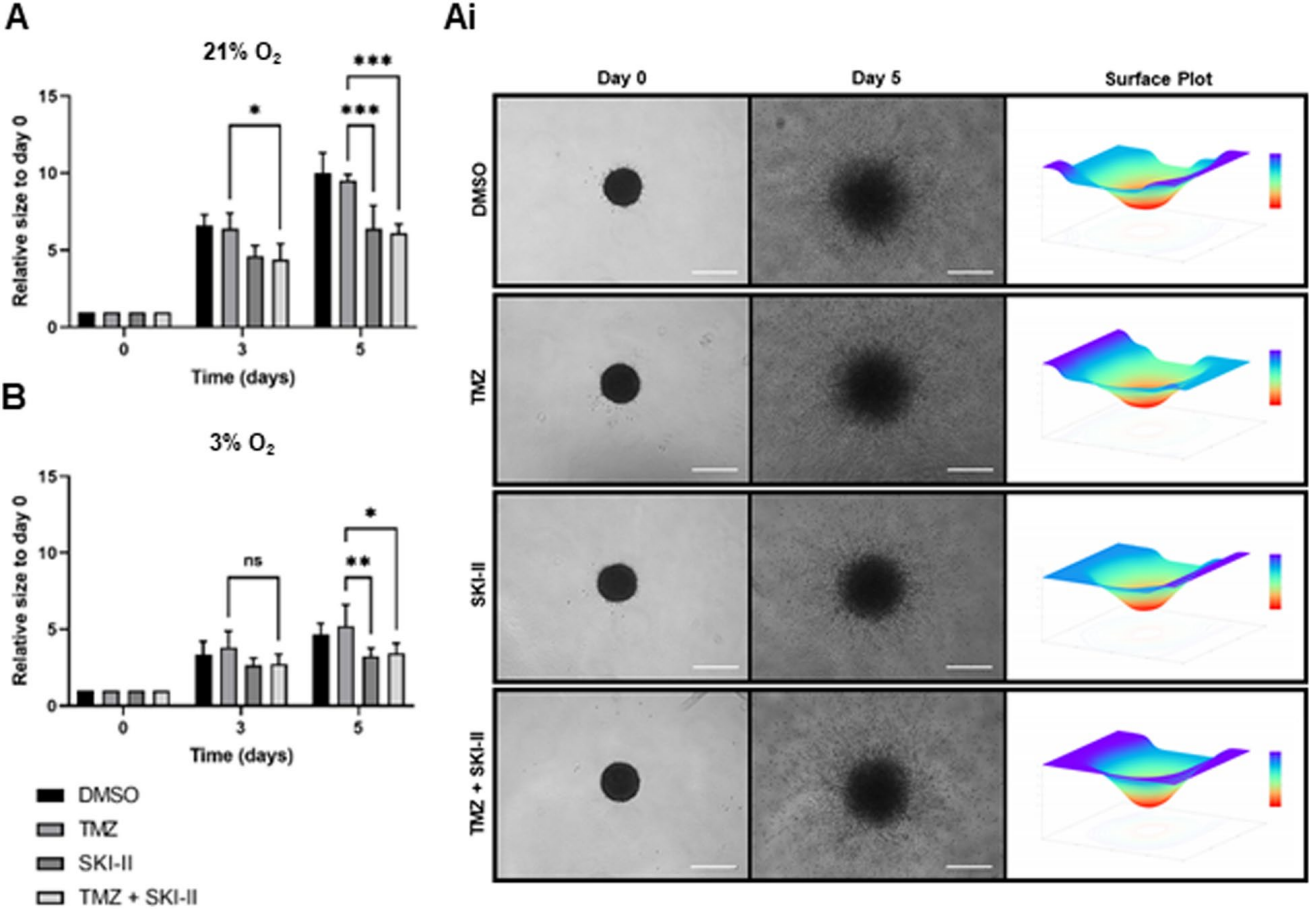
SKI-II alone and in combination with temozolomide (TMZ) impairs invasion of MGMT-positive glioblastoma cells. **A**, **B** The spheroid invasion assay was performed with U3054 cells (mesenchymal GB) treated with vehicle control (DMSO), 48 µM TMZ, 2.66 µM SKI-II, and the combination (TMZ + SKI-II) for up to 5 days under normoxia (21% O_2_) and hypoxia (3% O_2_). Quantification of the invaded area was performed using the Spheroid Analyzer (CLADIAC) as described in Material and Methods. (Ai) Light microscopy images of treated U3054 spheroids and respective surface plots. Data represent mean (± SD) of *n* = 3. Two-way ANOVA followed by Tukey’s multiple comparisons test was performed for statistical analysis. Scale bar = 500 µm

Next, we analyzed the effect of the combination on the invasion capacity of the human GB U3054 cells under normoxia and hypoxia, using a 3D-spheroid model. Cell arrangement as spheroids allows to better mimic in vivo conditions, such as formation of gradients of nutrients, oxygen and catabolites, that cannot be recapitulated in monolayer cultures. The U3054 line belongs to the transcriptional mesenchymal subtype and is derived from a recurrent GB [25]. Mesenchymal GB is considered to have an increased invasive potential and patients have a poorer prognosis [42, 43]. U3054 cells are MGMT-positive (Additional file 13), suggesting that these cells are resistant to TMZ therapy [44]. Indeed, as shown in Fig. 11, TMZ treatment did not affect the invasion capacity of U3054 spheroids under hypoxia and normoxia. In contrast, treatment with SKI-II alone and in combination reduced invasion by about 30 to 40% after 5 days under hypoxia and normoxia, respectively. Hypoxia per se reduced the invasion capacity of the U3054 cells in the collagen matrix.

## Discussion

Two notorious road-blocks to effective glioblastoma therapy are triggered by low oxygen condition, a hallmark of tumors: chemotherapy resistance and expansion of glioblastoma stem-like cells. In this in vitro study, we demonstrate that combining a clinically relevant dose of TMZ (48 µM) with SKI-II, an inhibitor of three enzymes of the sphingolipid metabolism, can alleviate these blocks.

The hypoxia-induced resistance to TMZ in GB cells was effectively overcome by the combination as demonstrated by the combination index analysis. The latter shows that treatment with 48 µMTMZ combined with 2.66 µM SKI-II (D2 combination, Table 1) under hypoxia allowed to drastically reduce the dose of TMZ that would have been necessary to achieve the same high efficiency if TMZ would have been used as single treatment. TMZ in combination with SKI-II was more effective than TMZ single treatment in inhibiting cell growth, inducing cell death, reducing invasion of differentiated GB cells (NCH82 cells) and impairing the self-renewal of GSCs (DMSO-1080 and TMZ-1080 cells) under hypoxia. SKI-II alone was as effective as in combination in impairing cell invasion of GB cells (U3054 cells) and GSC self-renewal, but not in inhibiting cell growth (as shown by synergism in combination) and inducing cell death (as shown by enhancement in combination). This demonstrates that in combination we leverage the cytotoxic and cytostatic capabilities of TMZ.

Studies have shown that the serum concentration of TMZ is in the range of 20–70 µM [21], whereas the intratumoral TMZ concentration is in the range of 3–35 µM [22]. In the present in vitro study, we could directly compare the cytotoxic effects of various combinations of doses of TMZ and SKI-II on the NCH82 cell line and we made the following observations. The combination used throughout our study (referred to as D2 in Table 1) was slightly more efficient than the D3 combination (96 µM TMZ and 2.66 µM SKI-II), indicating no advantage of using a higher TMZ dose in NCH82 cells. Combinations with even a very low TMZ dose were able to cause major cytotoxic effects in GB cells: the D1 combination (24 µM TMZ and 2.66 µM SKI-II) induced similar effects as the D2 combination (48 µM TMZ and 2.66 µM SKI-II) and with a comparable degree of synergy. NCH82 cells have low MGMT activity (11.5 ± 0.3 (S.D) fmol/mg) compared to other GB cell lines such as the LN18 (249 ± 10.6 (S.D) fmol/mg; our own unpublished data) and are wild-type for p53 [45]. Thus, lower doses of TMZ are expected to be more effective in these cells [46]. We showed that resistance to TMZ conferred by MGMT expression was also alleviated by the use of the combination, as demonstrated by the reduced invasion capacity of serum-free and MGMT-positive U3054 cells. This reduced capacity could indicate a decrease in motility, or reflect reduced proliferation and/or increased cell death. Altogether the data suggest that using TMZ in combination at half of the dose similar to that used in the clinic (100 µM) might result in an increased therapeutic efficiency in GB cells with (high) MGMT activity.

As already mentioned, a striking feature of the combination is its capacity to impair self-renewal of the two GSC lines we tested and particularly that of TMZ-resistant GSCs under hypoxia. The potential of a cell for self-renewal is taken as a measure of its stemness. Impairment of this biological state can lead to a reduced resistance of cancer stem cells to therapy. The combination however did not exhibit, or only weakly, cytotoxic effects to the DMSO-1080 and TMZ-1080 GSC cell lines. Notwithstanding this observation, we cannot rule out the possibility that the combination, by altering sphingolipid metabolism, increases their sensitivity to chemotherapeutics other than TMZ as a result of the reduction of their stemness status. This certainly would be worth to investigate.

In this study we used the SKI-II inhibitor to alter sphingolipid metabolism. As already mentioned, SKI-II was first discovered as a specific dual inhibitor of SK1 and SK2 with a Ki of 16 µM for SK1 [14, 16]. A few years later, Cingolani et al. [15] provided evidence that SKI-II inhibits the desaturase 1, a very important enzyme of the de novo sphingolipid pathway, with a Ki (0.3 µM). The use of 10 µM SKI-II in our previous study led to a significant increase in dihydrosphingolipids levels and a strong decrease in S1P production, in line with the capacity of the inhibitor to affect, at that concentration, the enzymatic activities of both SK1 and DES1 [17]. In the present study, treatment with 2.66 µM SKI-II did not cause these alterations. Our data rather suggest that SKI-II at this low concentration mostly affects the desaturase as indicated by the reduced ratio of ceramide/dihydrosphingosine in both oxygen conditions, and leads to ceramide decrease. It only mildly, if at all, affects the sphingosine kinase, leading to modest decrease in S1P. The comparable profile of the levels in ceramide and its metabolites after treatments suggests that the metabolic flux between these sphingolipids is not affected and that the S1P decrease can be interpreted as the result of the lower amount of ceramide available for sphingosine generation. On the basis of these observations, we conclude that SKI-II at 2,66 µM, alone and in combination, mainly affects the desaturase and not the sphingosine kinase. The partial depletion of S1P did not hinder the anti-tumor effects of the low-dose combination and might even be beneficial for cells within the parenchyma, including astrocytes, neurons and tumor-associated cells. Extracellular S1P release by dying cells was reported to serve as a signal for macrophages to phagocytose these dying cells [47]. Suppression of sphingosine kinase in activated microglia was reported to decrease their pro-inflammatory activities [48], which are desirable to eliminate tumor cells. Thus, strategies that would preserve a low level of SK activity, such as the use of low SKI-II doses, might contribute to an environment that is not only protective to healthy cells, but also supportive to immune cells through the facilitation of their phagocytic and pro-inflammatory response. This suggests that the combination might be applied together with immunotherapies designed to promote the anti-tumor activities of tumor-associated microglia/macrophages.

Oxidative stress seems to have an essential role in cell death induced by the low-dose (present study) and the high-dose (our previous study [18]) combination of TMZ and SKI-II, as shown by their peroxynitrite-dependent effects (e.g. ER stress and viability). However, whereas the NCH82 cells treated with the high-dose combination increased their autophagic flux and died by caspase-dependent apoptosis [18], cells treated with the low-dose combination showed no alteration in the autophagic flux and polarization of mitochondria and died through a caspase-independent, potentially non-apoptotic, form of cell death. The lack of accumulation of dihydrosphingolipids in the low-dose combination could correlate with the absence of changes in the autophagic flux. Several studies report autophagy induction by dihydrosphingosine and dihydroceramide [49]. Apoptosis and autophagy were shown to be induced as a linear function of TMZ dose when MGMT is lacking [50]. Therefore, the difference in the dose of TMZ used in our studies (48 µM vs 500 µM) have likely contributed to the dissimilarities observed in autophagy and cell death mechanisms. The lack of PI-positive/Annexin V-negative NCH82 cells suggests that membrane integrity is kept and primary necrosis can be ruled out. The increase in the percentage of Annexin V-positive cells over time in all conditions (except in the vehicle control) demonstrates that phosphatidylserine (PS) exposure is a morphological feature induced by SKI-II and TMZ. Exposure of PS is mediated via scramblases, which are activated by caspase 3/7 cleavage. However, caspase-independent mechanisms of PS exposure were also described in non-apoptotic forms of cell death [51]. Namely, PS exposure was shown to occur due to sustained increase in the concentration of cytoplasmic calcium in a form of paraptosis [52]. The appearance of extensive cytoplasmic vacuolization, ER dilation, and lack of caspase activation and mitochondrial depolarization suggests that the low-dose (TMZ + SKI-II) combination could induce a paraptosis-like cell death in NCH82 cells [53]. Although plausible, this conclusion is a hypothesis that will have to be proven.

The mechanism underlying oxidative stress induction by SKI-II in our experimental system is unclear. Sphingolipids are able to regulate cellular redox homeostasis: ceramide can activate NADPH oxidase, supporting peroxynitrite formation [54]; whereas, glucosylceramide can inhibit it [55]. We observed a decrease of C16- and C24-ceramide, ceramide-1-phosphate and sphingomyelin in both oxygen conditions. A decreased level of C16-ceramide has been related to ER stress induction as it can promote Ca2 + release from the ER and activate the ATF6/CHOP axis [56]. S1P, which is still produced under treatment with the combination, might contribute to oxidative stress through interaction with its ligand, S1PR1. Indeed, activation of S1PR1 was reported to support peroxynitrite formation by increasing the generation of NADPH oxidase-derived superoxide and nitric oxide synthase-derived nitric oxide [57]. The role of S1P and its receptor in the response of the tumor cells to the combination remains to be elucidated.

## Conclusion

Experience of the past decades in delineating cancer therapeutic strategies illustrates that focusing on one singular target is insufficient and ineffective, namely in tumors as complex as glioblastoma. In the present study, we wanted to explore the benefit of combining two drugs affecting two different biological processes in order to demonstrate at a preclinical level the value of the sphingolipid metabolism as a target for cancer therapy, alone and in combination with temozolomide, the first-line agent in glioblastoma therapy. Given their key role in controlling cell fate, sphingosine kinases are prime targets to modulate sphingolipid metabolism [10] and have been the focus of many in vitro and in vivo studies in the past twenty years [58, 59]. However, SK inhibitors have scarcely been implemented in clinical trials and, to paraphrase Gault et al. [58], they are “still benched on (their) way to the bedside”. One explanation may lie in the contradictory data that have been reported on the cytotoxic activities of SK inhibitors on various tumor cell models, thus diminishing their potential value. We hope, with the present data, to rekindle interest for SK inhibitors, and, beyond SK inhibitors, for the sphingolipid metabolism as a relevant and attractive therapeutic target. Indeed, we provide evidence that modulation of sphingolipid metabolism, combined with the use of TMZ, has the potential to affect various biological functions of differentiated and stem glioblastoma cells under atmospheric and low oxygen concentration. These observations warrant further experimental exploration to assess the clinical potential of such a combination to reduce the glioblastoma stem cell population in tumors and prevent relapse.

### Supplementary Information

**Additional file 1.** LC and MS conditions for sphingolipid analysis by liquid chromatography/multiple reaction monitoring (LC/MRM). LC-MS gradient and solvent system are depicted in the table below. The MS ion source parameters were as follows: curtain gas was 40 psi; source heater temperature 550 °C; ion spray voltage was set to−4500 V in negative ion mode and to +5200 V in positive ion mode.

**Additional file 2.** Sphingolipid analysis by liquid chromatography/multiple reaction monitoring (LC/MRM). MRM transitions and parameters used in positive ion mode for sphingolipid profile and quantification are depicted.

**Additional file 3.** Calculation of the median-effect dose. The median-effect dose (ED50) of temozolomide (TMZ) and the sphingosine kinase inhibitor SKI-II was calculated in NCH82 cells at normoxia (21% O_2_) and hypoxia (3% O_2_) for 5 days using the median-effect analysis program CompuSyn (Nick Martin, MIT, Cambridge, MA, 2005).

**Additional file 4.** Combination design of temozolomide (TMZ) and the sphingosine kinase inhibitor SKI-II. Fifteen (TMZ + SKI-II) combinations were made based on the ED50 of each drug (SKI-II ED50 = 1.33 µM; TMZ ED50 = 96 µM).

**Additional file 5. **Dose Reduction Index (DRI) of combinations of temozolomide (TMZ) and the sphingosine kinase inhibitor SKI-II at 21% and 3% O_2_ in NCH82 cells. The dose (µM) of TMZ and SKI-II and respective DRI for each combination was calculated using CompuSyn software. DRI=1, >1, and <1 indicates no dose-reduction, favorable dose-reduction, and not favorable dose-reduction, respectively, for each drug in the combination.

**Additional file 6.** Full-length blots of cleaved caspase-3 and GAPDH detection shown in Fig. 3 A. After the protein transfer, the nitrocellulose membrane was cut below the 30 kDa marker, and each strip was incubated with antibodies against GAPDH or Cleaved Caspase-3. Left panel (21 % O_2_): exposure times were 2 min for GAPDH, 5 min for Cleaved Caspase-3. Right panel (3% O_2_): exposure times were 2 min for GAPDH, 5 min for Cleaved Caspase-3.

**Additional file 7.** The combination does not affect autophagic flux in glioblastoma cells. NCH82 cells were treated with 48 µM temozolomide (TMZ) combined with 2.66 µM SKI-II (TMZ+SKI-II) in the presence and absence of 100 nM Bafilomycin A1 (BA1) under 21% O_2_ and 3% O_2_. (A, B) Cell extracts were separated on SDS- PAGE and transferred on nitrocellulose membrane. DMSO and (TMZ+SKI-II) gels were run in parallel. Representative blots are shown. The expression levels of p62 (Ai, Bi) and LC3-II (Aii, Bii) were quantified and normalized to GAPDH. Autophagic flux under basal conditions (DMSO) and under treatment (TMZ + SKI-II) was determined by the subtraction of LC3-II levels without BA1 (Ctrl) from LC3-II levels with BA1 (Aiii, Biii). LC3-II (*n* = 3); p62 (*n* = 2). The results are shown as mean (±SD). Two-way ANOVA followed by Tukey’s multiple comparisons test was performed for statistical analysis. Full-length blots are presented in Additional file 8.

**Additional file 8.** Full-length blots of LC3, p62 and GAPDH detection shown in Additional file 7 A (panel A) and Additional file 7 B (panel B). After the protein transfer, the nitrocellulose membrane was cut in three pieces, below the 50 and 30 kDa markers and each strip was incubated with antibodies against p62, GAPDH or LC3B. Panel A, left (DMSO) and right (TMZ + SK-II): exposure times were p62, 3 min (left and right); GAPDH, 10 sec (left) and 30 sec (right); LC3B, 4 min (left and right). Panel B, left (DMSO) and right (TMZ + SK-II): exposure times were p62, 4 min (left) and 3 min (right); GAPDH, 1 min (left) and 30 sec (right); LC3B, 4 min (left and right).

**Additional file 9.** Full-length blots of BiP/GRP78 and GAPDH detection shown in Fig. 6 A. Left (21 % O_2_) and right (3 % O_2_) panels: After the protein transfer, the nitrocellulose membrane was cut in two pieces, below the 50 kDa marker, and each strip was incubated with antibodies against GAPDH or BiP/GRP78. Exposure times were: GAPDH 1 min (left and right); BiP/GRP78 1 min (left and right).

**Additional file 10.** Full-length blots of BiP/GRP78 and GAPDH detection shown in Fig.6 C. After the protein transfer, the nitrocellulose membrane was cut in two pieces, between the 60 kDa and the 50 kDa markers and each strip was incubated with antibodies against GAPDH or BiP/GRP78. Exposure times were: GAPDH 20 sec; BiP/GRP78 1 min.

**Additional file 11.** ELDA analysis: Full data files.

**Additional file 12.** Immunofluorescence detection of activated caspase-3 in treated GSC. DMSO-1080 and TMZ-1080 GSC cells plated on ornithin-coated glass coverslips were treated with 2.66 µM SKI-II, 48 µM temozolomide (TMZ) and the combination (TMZ+SKI-II) in triplicates. After 5 days of incubation, cells were fixed with 4% paraformaldehyde and stained for cleaved caspase-3, nestin and Dapi. (A) Quantification of cleaved caspase-3 positive cells. Data are presented as percent of total cells counted in 7 to 9 regions of triplicates, and for a total number of cells of 700 to 1100. (B) Representative images of apoptotic cells in 1080-TMZ cells treated with SKI-II under normoxia (left) or hypoxia (right). Magnification 40X, scale bar 50 µM. Left panels in each composite picture: enlarged images of apoptotic cells (marked by arrows in the right panels) manifesting morphological hallmarks of apoptosis such as chromatin condensation and cytoplasmic disintegration. *n* = 1.

**Additional file 13.** The U3054 cells express the O6-methylguanine-DNA methyltransferase.

## Data Availability

The datasets used and/or analysed during the current study are available from the corresponding author on reasonable request.

## Abbreviations

AV: Annexin-V
BA1: Bafilomycin A1
C1P: Ceramide-1-phosphate
CPT: Camptothecin
DES1: Dihydroceramide desaturase 1
DRI: Dose-reduction index
ED50: Median effect dose
ELDA: Extreme limiting dilution analysis
ER: Endoplasmic reticulum
GB: Glioblastoma
GSC: Glioblastoma stem cell
Ki: Inhibition constant
MGMT: O6-methylguanine-DNA methyltransferase
PI: Propidium iodide
PS: Phosphatidylserine
ROS: Reactive oxygen species
S1P: Sphingosine-1-phosphate
S1PR1: S1P receptor 1
SK: Sphingosine kinase
SM: Sphingomyelin
SRB: Sulforhodamine B
TMZ: Temozolomide
UPR: Unfolded protein response
zVAD: Z-VAD-FMK
