# Supplementary material for: Targeting sphingolipid metabolism with the sphingosine kinase inhibitor SKI-II overcomes hypoxia-induced chemotherapy resistance in glioblastoma cells: effects on cell death, self-renewal, and invasion
Source: BMC Cancer. 2023 Aug 16;23:762. doi: 10.1186/s12885-023-11271-w (PMC10433583; doi:10.1186/s12885-023-11271-w)
Supplement: Supplementary file 1 — Additional file 1. LC and MS conditions for sphingolipid analysis by liquid chromatography/multiple reaction monitoring (LC/MRM). LC-MS gradient and solvent system are depicted in the table below. The MS ion source parameters were as follows: curtain gas was 40 psi; source heater temperature 550 °C; ion spray voltage was set to−4500 V in negative ion mode and to +5200 V in positive ion mode. [file 12885_2023_11271_MOESM1_ESM.pdf]

Additional files

Additional File 1 - LC and MS conditions for sphingolipid analysis by liquid chromatography/multiple reaction monitoring (LC/MRM). LC-MS gradient and solvent system are depicted in the table below. The MS ion source parameters were as follows: curtain gas was 40 psi; source heater temperature 550 °C; ion spray voltage was set to −4500 V in negative ion mode and to +5200 V in positive ion mode.

Total time (min)	Flow rate (µL/min)	Solvent A %	Solvent B %
0	200	60	40
3	200	60	40
45	200	10	90
46	200	1	99
53	200	1	99
55	200	60	40

Solvent A	methanol/water (1:1; v/v) containing 7.5 mM ammonium formate and 0.1% triethylamine
Solvent B	methanol/isopropanol (2:8; v/v) containing 7.5 mM ammonium formate and 0.1% triethylamine
