# Supplementary material for: Targeting sphingolipid metabolism with the sphingosine kinase inhibitor SKI-II overcomes hypoxia-induced chemotherapy resistance in glioblastoma cells: effects on cell death, self-renewal, and invasion
Source: BMC Cancer. 2023 Aug 16;23:762. doi: 10.1186/s12885-023-11271-w (PMC10433583; doi:10.1186/s12885-023-11271-w)
Supplement: Supplementary file 2 — Additional file 2. Sphingolipid analysis by liquid chromatography/multiple reaction monitoring (LC/MRM). MRM transitions and parameters used in positive ion mode for sphingolipid profile and quantification are depicted. [file 12885_2023_11271_MOESM2_ESM.pdf]

Additional File 2 - Sphingolipid analysis by liquid chromatography/multiple reaction monitoring (LC/MRM). MRM transitions and parameters used in positive ion mode for sphingolipid profile and quantification are depicted.

Type	Q1 mass (Da)	Q3 mass (Da)	Dwell time (ms)	Name	DP (V)	CE (V)	CXP (V)
calibrant	300,28	252,3	10	SPH C18:1	60	22	26
Internal standard (ISTD)	286,47	268,2	10	SPH 17 ISTD	60	22	26
calibrant	536,5	262,3	10	CER 16	30	34	26
calibrant	648,63	262,3	10	CER 24	30	34	26
ISTD	552,54	264,4	10	CER 17 ISTD	30	34	26
quantifier	538,5	264,2	10	CER d18:1/16:0 quan	30	34	26
quantifier	648,5	264,2	10	CER d18:1/24:1 quan	30	34	26
quantifier	815,7	184,073	10	SM d18:1/24:0	50	40	26
quantifier	703,575	184,073	10	SM d18:1/16:0	50	40	26
quantifier	382,25	266,2	10	dhS1P C18:0	30	30	26
qualifier	302,28	284,2	10	dhSPH 18:0 (H2O)	60	22	26
quantifier	302,28	266,2	10	dhSPH 18:0	60	22	26
qualifier	540,5	266,2	10	dhCer 18:0/16:0	30	34	26
quantifier	540,5	522,4	10	dhCer 18:0/16:0 (H2O)	30	34	26
quantifier	650,5	266,2	10	dhCer 18:0/24:1	30	34	26
qualifier	650,5	632,5	10	dhCer 18:0/24:1 (H2O)	30	34	26
quantifier	566,5	264,3	10	dhCer 18:1/18:0	30	34	26
quantifier	568,6	266,3	10	dhCer 18:0/18:0	30	34	26
quantifier	733,6	184,073	10	SM d18:0/18:0	50	40	26
quantifier	817,7	184,073	10	SM d18:0/24:0	50	40	26
quantifier	705,575	184,073	10	SM d18:0/16:0	50	40	26
ISTD	647,51	184,073	10	SM ISTD	50	40	26
calibrant	731,61	184,073	10	SM cal d18:1/18:0	50	40	26
quantifier	646,49	264,4	10	dhC1P 18:1	40	40	26
quantifier	648,5	266,4	10	dhC1P 18:0	40	40	26

Abbreviations: DP, Declustering Potential ; CE, Collision Energy ; CXP, Collision Cell Exit Potential.
