# Supplementary material for: Targeting sphingolipid metabolism with the sphingosine kinase inhibitor SKI-II overcomes hypoxia-induced chemotherapy resistance in glioblastoma cells: effects on cell death, self-renewal, and invasion
Source: BMC Cancer. 2023 Aug 16;23:762. doi: 10.1186/s12885-023-11271-w (PMC10433583; doi:10.1186/s12885-023-11271-w)
Supplement: Supplementary file 3 — Additional file 3. Calculation of the median-effect dose. The median-effect dose (ED50) of temozolomide (TMZ) and the sphingosine kinase inhibitor SKI-II was calculated in NCH82 cells at normoxia (21% O2) and hypoxia (3% O2) for 5 days using the median-effect analysis program CompuSyn (Nick Martin, MIT, Cambridge, MA, 2005). [file 12885_2023_11271_MOESM3_ESM.pdf]

Additional File 3 – Calculation of the median-effect dose. The median-effect dose (ED50) of temozolomide (TMZ) and the sphingosine kinase inhibitor SKI-II was calculated in NCH82 cells at normoxia (21% O_2) and hypoxia (3% O_2) for 5 days using the median-effect analysis program CompuSyn (Nick Martin, MIT, Cambridge, MA, 2005).

Compound	Concentration range (μM)	21% O_2			3% O_2		
		ED50 (μM)	*m*	*r*	ED50 (μM)	*m*	*r*
TMZ	2.5 - 640	95.67	-0.358	-0.994	427.95	-0.376	-0.976
SKI-II	0.156 - 10	1.33	-0.887	-0.992	1.36	-0.934	-0.992
