# Supplementary material for: Targeting sphingolipid metabolism with the sphingosine kinase inhibitor SKI-II overcomes hypoxia-induced chemotherapy resistance in glioblastoma cells: effects on cell death, self-renewal, and invasion
Source: BMC Cancer. 2023 Aug 16;23:762. doi: 10.1186/s12885-023-11271-w (PMC10433583; doi:10.1186/s12885-023-11271-w)
Supplement: Supplementary file 4 — Additional file 4. Combination design of temozolomide (TMZ) and the sphingosine kinase inhibitor SKI-II. Fifteen (TMZ + SKI-II) combinations were made based on the ED50 of each drug (SKI-II ED50 = 1.33 µM; TMZ ED50 = 96 µM). [file 12885_2023_11271_MOESM4_ESM.pdf]

Additional File 4 - Combination design of temozolomide (TMZ) and the sphingosine kinase inhibitor SKI-II. Fifteen (TMZ + SKI-II) combinations were made based on the ED50 of each drug (SKI-II ED50 = 1.33 µM; TMZ ED50 = 96 µM).

**Drug Combination (SKI-II + TMZ)**		**TMZ**		
		0.25 x ED50	0.5 x ED50	1 x ED50
	0.25 x ED50	A1	A2	A3
	0.5 x ED50	B1	B2	B3
**SKI-II**	1 x ED50	C1	C2	C3
	2 x ED50	D1	D2	D3
	4 x ED50	E1	E2	E3
