# Supplementary material for: Targeting sphingolipid metabolism with the sphingosine kinase inhibitor SKI-II overcomes hypoxia-induced chemotherapy resistance in glioblastoma cells: effects on cell death, self-renewal, and invasion
Source: BMC Cancer. 2023 Aug 16;23:762. doi: 10.1186/s12885-023-11271-w (PMC10433583; doi:10.1186/s12885-023-11271-w)
Supplement: Supplementary file 5 — Additional file 5. Dose Reduction Index (DRI) of combinations of temozolomide (TMZ) and the sphingosine kinase inhibitor SKI-II at 21% and 3% O2 in NCH82 cells. The dose (µM) of TMZ and SKI-II and respective DRI for each combination was calculated using CompuSyn software. DRI=1, >1, and <1 indicates no dose-reduction, favorable dose-reduction, and not favorable dose-reduction, respectively, for each drug in the combination. [file 12885_2023_11271_MOESM5_ESM.pdf]

Additional File 5 - Dose Reduction Index (DRI) of combinations of temozolomide (TMZ) and the sphingosine kinase inhibitor SKI-II at 21% and 3% O_2 in NCH82 cells. The dose (µM) of TMZ and SKI-II and respective DRI for each combination was calculated using CompuSyn software. DRI=1, >1, and <1 indicates no dose-reduction, favorable dose-reduction, and not favorable dose-reduction, respectively, for each drug in the combination.

Drug Combi-nation	21% O_2				3% O_2			
	Dose TMZ	Dose SKI-II	DRI TMZ	DRI SKI-II	Dose TMZ	Dose SKI-II	DRI TMZ	DRI SKI-II
A1	119.56	1.45	4.98	4.41	56.87	0.60	2.36	1.83
A2	454.20	2.49	9.46	7.56	234.52	1.06	4.88	3.23
A3	1060.29	3.51	11.04	10.66	406.25	1.33	4.23	4.04
B1	296.49	2.10	12.35	3.18	171.07	0.94	7.12	1.42
B2	544.37	2.68	11.34	4.07	275.44	1.14	5.73	1.72
B3	1213.44	3.71	12.64	5.63	354.22	1.26	3.68	1.91
C1	311.70	2.14	12.98	1.61	443.22	1.38	18.46	1.03
C2	722.46	3.01	15.05	2.26	573.87	1.53	11.95	1.15
C3	915.60	3.31	9.53	2.49	674.22	1.63	7.02	1.23
D1	2745.14	5.16	114.38	1.94	9114.44	4.67	379.76	1.75
D2	4041.18	6.04	84.19	2.27	12113.0	5.24	252.35	1.97
D3	5543.66	6.86	57.74	2.58	17842.0	6.12	185.85	2.30
E1	16124.8	10.57	671.86	1.98	70426.7	10.65	2934.44	2.00
E2	17400.5	10.91	362.51	2.05	58745.6	9.90	1223.87	1.86
E3	18352.1	11.14	191.16	2.09	68447.6	10.53	712.99	1.97
