# Supplementary material for: Targeting sphingolipid metabolism with the sphingosine kinase inhibitor SKI-II overcomes hypoxia-induced chemotherapy resistance in glioblastoma cells: effects on cell death, self-renewal, and invasion
Source: BMC Cancer. 2023 Aug 16;23:762. doi: 10.1186/s12885-023-11271-w (PMC10433583; doi:10.1186/s12885-023-11271-w)

Additional File 6 - Full-length blots of cleaved caspase-3 and GAPDH detection shown in Figure 3 A.

After the protein transfer, the nitrocellulose membrane was cut below the 30 kDa marker, and each strip was incubated with antibodies against GAPDH or Cleaved Caspase-3.

Left panel (21 % O_2): exposure times were 2 min for GAPDH, 5 min for Cleaved Caspase-3.

Right panel (3% O_2): exposure times were 2 min for GAPDH, 5 min for Cleaved Caspase-3.

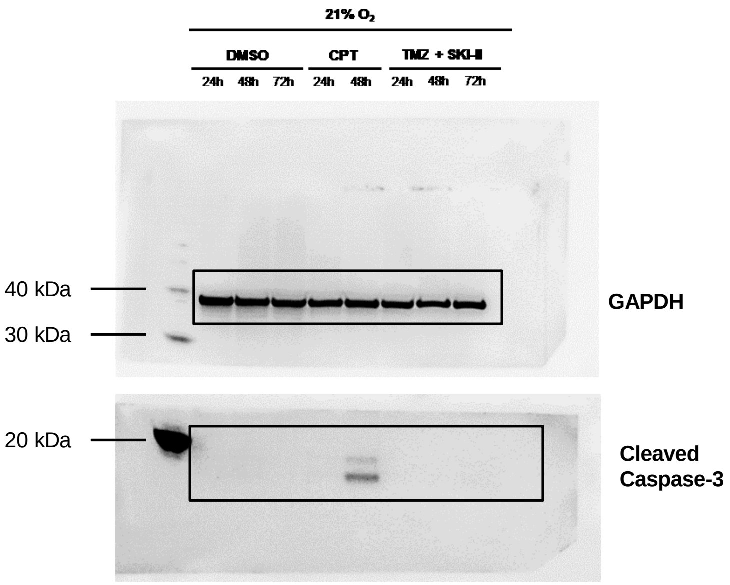

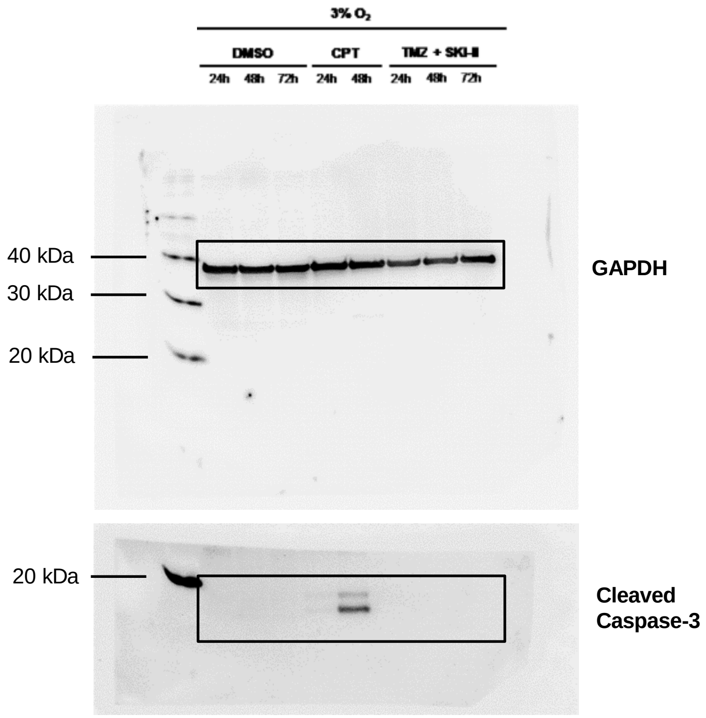

Supplement: Supplementary file 6 — Additional file 6. Full-length blots of cleaved caspase-3 and GAPDH detection shown in Fig. 3 A. After the protein transfer, the nitrocellulose membrane was cut below the 30 kDa marker, and each strip was incubated with antibodies against GAPDH or Cleaved Caspase-3. Left panel (21 % O2): exposure times were 2 min for GAPDH, 5 min for Cleaved Caspase-3. Right panel (3% O2): exposure times were 2 min for GAPDH, 5 min for Cleaved Caspase-3. [file 12885_2023_11271_MOESM6_ESM.pdf]
