# Supplementary material for: Targeting sphingolipid metabolism with the sphingosine kinase inhibitor SKI-II overcomes hypoxia-induced chemotherapy resistance in glioblastoma cells: effects on cell death, self-renewal, and invasion
Source: BMC Cancer. 2023 Aug 16;23:762. doi: 10.1186/s12885-023-11271-w (PMC10433583; doi:10.1186/s12885-023-11271-w)

Additional File 7 - The combination does not affect autophagic flux in glioblastoma cells. NCH82 cells were treated with 48 µM temozolomide (TMZ) combined with 2.66 µM SKI-II (TMZ+SKI-II) in the presence and absence of 100 nM Bafilomycin A1 (BA1) under 21% O_2 and 3% O_2. (A, B) Cell extracts were separated on SDS-PAGE and transferred on nitrocellulose membrane. DMSO and (TMZ+SKI-II) gels were run in parallel. Representative blots are shown. The expression levels of p62 (Ai, Bi) and LC3-II (Aii, Bii) were quantified and normalized to GAPDH. Autophagic flux under basal conditions (DMSO) and under treatment (TMZ + SKI-II) was determined by the subtraction of LC3-II levels without BA1 (Ctrl) from LC3-II levels with BA1 (Aiii, Biii). LC3-II (n = 3); p62 (n = 2). The results are shown as mean (±SD). Two-way ANOVA followed by Tukey's multiple comparisons test was performed for statistical analysis. Full-length blots are presented in Additional file 8.

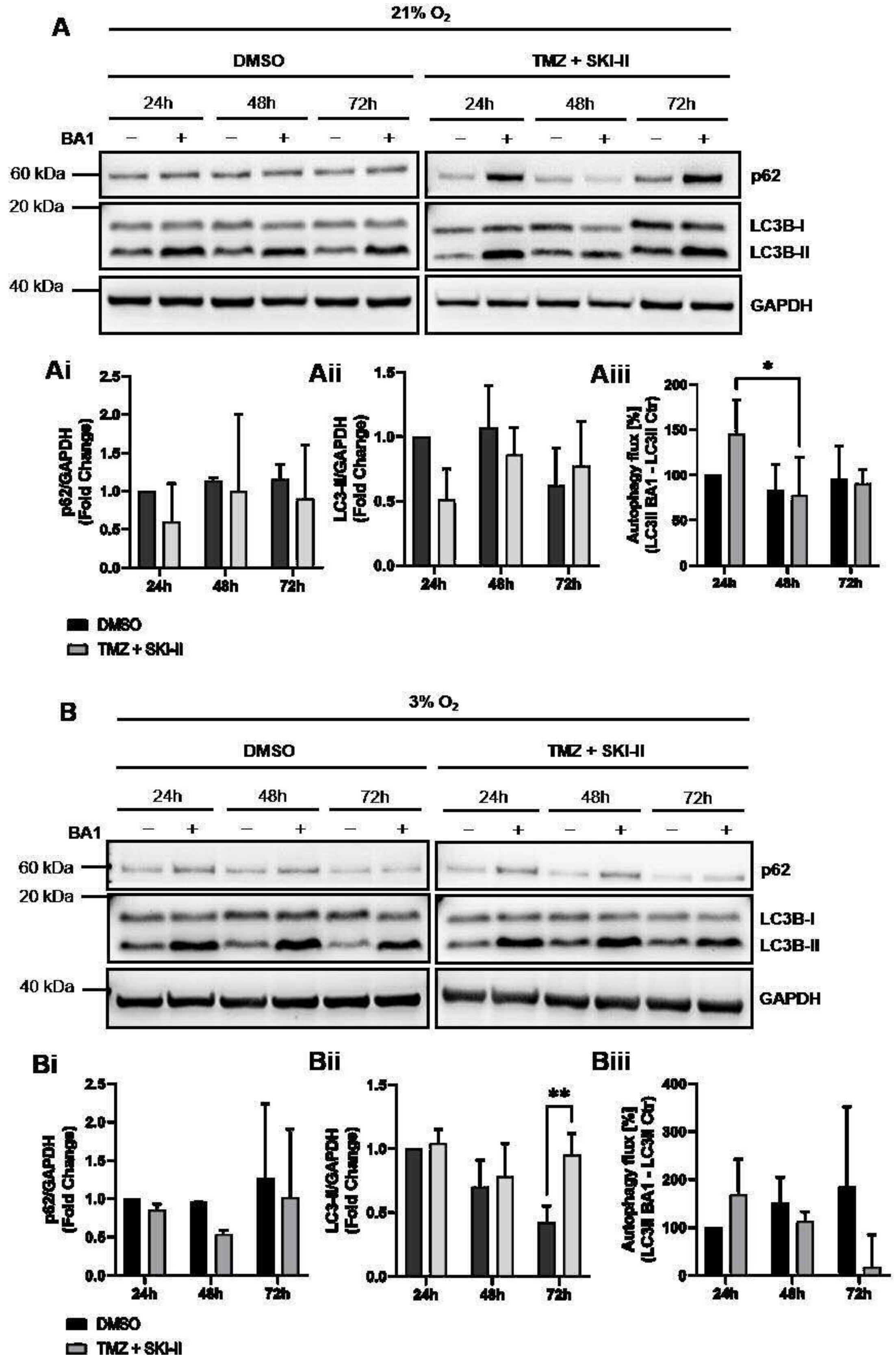

Supplement: Supplementary file 7 — Additional file 7. The combination does not affect autophagic flux in glioblastoma cells. NCH82 cells were treated with 48 µM temozolomide (TMZ) combined with 2.66 µM SKI-II (TMZ+SKI-II) in the presence and absence of 100 nM Bafilomycin A1 (BA1) under 21% O2 and 3% O2. (A, B) Cell extracts were separated on SDS- PAGE and transferred on nitrocellulose membrane. DMSO and (TMZ+SKI-II) gels were run in parallel. Representative blots are shown. The expression levels of p62 (Ai, Bi) and LC3-II (Aii, Bii) were quantified and normalized to GAPDH. Autophagic flux under basal conditions (DMSO) and under treatment (TMZ + SKI-II) was determined by the subtraction of LC3-II levels without BA1 (Ctrl) from LC3-II levels with BA1 (Aiii, Biii). LC3-II (n = 3); p62 (n = 2). The results are shown as mean (±SD). Two-way ANOVA followed by Tukey’s multiple comparisons test was performed for statistical analysis. Full-length blots are presented in Additional file 8. [file 12885_2023_11271_MOESM7_ESM.pdf]
