# Supplementary material for: Targeting sphingolipid metabolism with the sphingosine kinase inhibitor SKI-II overcomes hypoxia-induced chemotherapy resistance in glioblastoma cells: effects on cell death, self-renewal, and invasion
Source: BMC Cancer. 2023 Aug 16;23:762. doi: 10.1186/s12885-023-11271-w (PMC10433583; doi:10.1186/s12885-023-11271-w)

Additional File 8 - Full-length blots of LC3, p62 and GAPDH detection shown in Additional File 7 A (panel A) and Additional File 7 B (panel B).

After the protein transfer, the nitrocellulose membrane was cut in three pieces, below the 50 and 30 kDa markers and each strip was incubated with antibodies against p62, GAPDH or LC3B.

Panel A, left (DMSO) and right (TMZ + SK-II): exposure times were p62, 3 min (left and right); GAPDH, 10 sec (left) and 30 sec (right); LC3B, 4 min (left and right).

Panel B, left (DMSO) and right (TMZ + SK-II): exposure times were p62, 4 min (left) and 3 min (right); GAPDH, 1 min (left) and 30 sec (right); LC3B, 4 min (left and right).

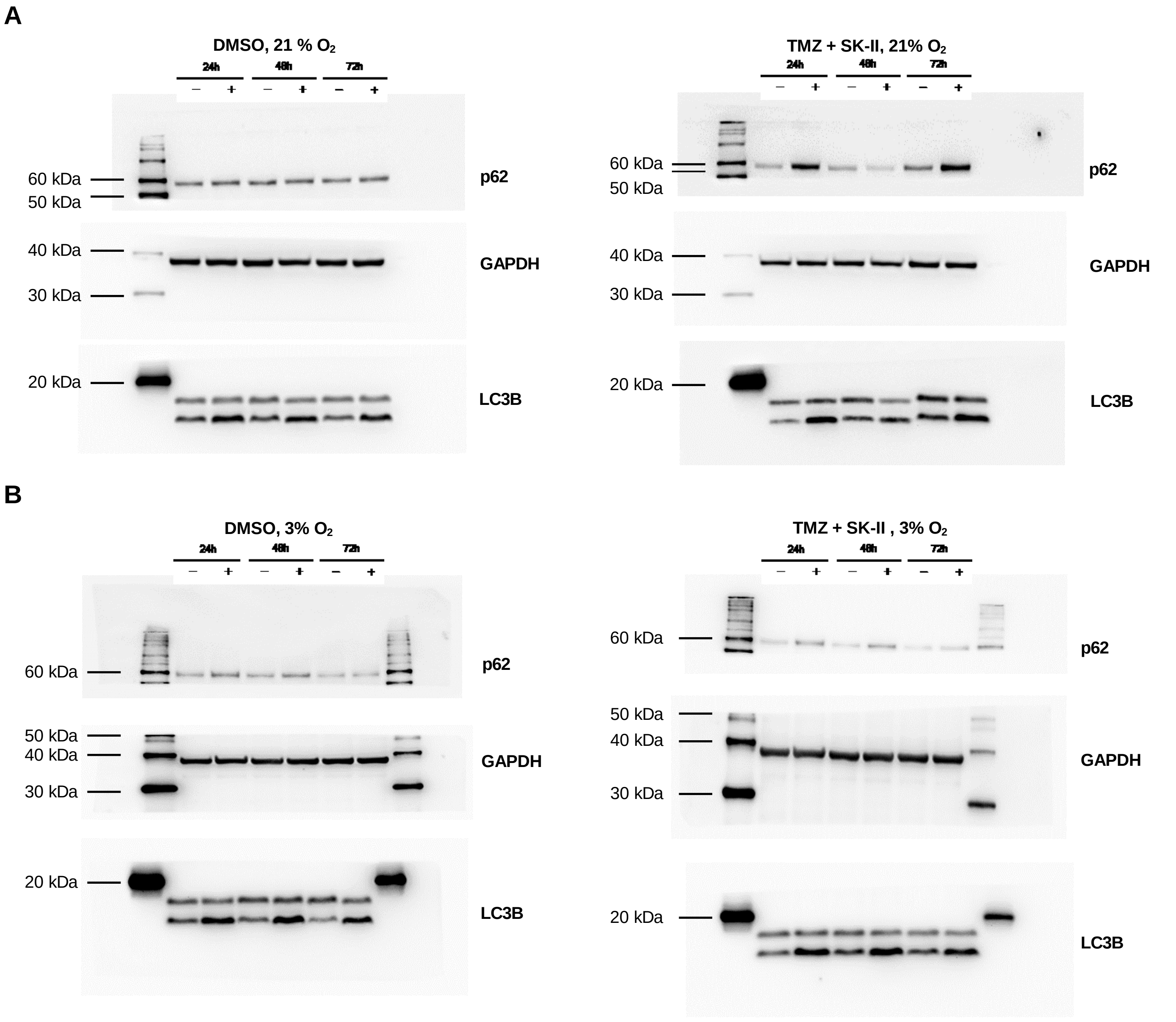

Supplement: Supplementary file 8 — Additional file 8. Full-length blots of LC3, p62 and GAPDH detection shown in Additional file 7 A (panel A) and Additional file 7 B (panel B). After the protein transfer, the nitrocellulose membrane was cut in three pieces, below the 50 and 30 kDa markers and each strip was incubated with antibodies against p62, GAPDH or LC3B. Panel A, left (DMSO) and right (TMZ + SK-II): exposure times were p62, 3 min (left and right); GAPDH, 10 sec (left) and 30 sec (right); LC3B, 4 min (left and right). Panel B, left (DMSO) and right (TMZ + SK-II): exposure times were p62, 4 min (left) and 3 min (right); GAPDH, 1 min (left) and 30 sec (right); LC3B, 4 min (left and right). [file 12885_2023_11271_MOESM8_ESM.pdf]
