# Supplementary material for: Targeting sphingolipid metabolism with the sphingosine kinase inhibitor SKI-II overcomes hypoxia-induced chemotherapy resistance in glioblastoma cells: effects on cell death, self-renewal, and invasion
Source: BMC Cancer. 2023 Aug 16;23:762. doi: 10.1186/s12885-023-11271-w (PMC10433583; doi:10.1186/s12885-023-11271-w)

Additional File 9 - Full-length blots of BiP/GRP78 and GAPDH detection shown in Figure 6 A.

Left (21 % O_2) and right (3 % O_2) panels: After the protein transfer, the nitrocellulose membrane was cut in two pieces, below the 50 kDa marker, and each strip was incubated with antibodies against GAPDH or BiP/GRP78. Exposure times were: GAPDH 1 min (left and right); BiP/GRP78 1 min (left and right).

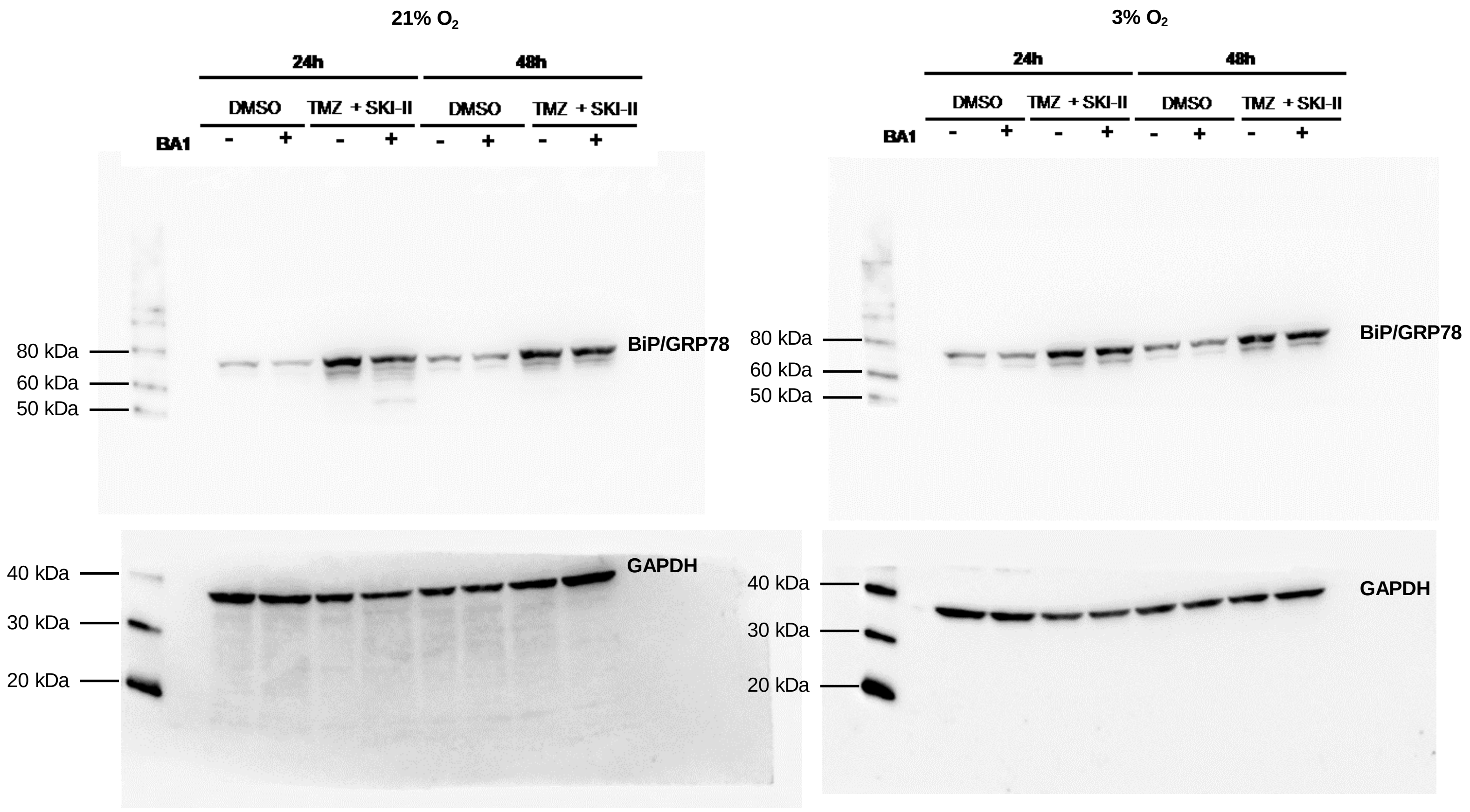

Supplement: Supplementary file 9 — Additional file 9. Full-length blots of BiP/GRP78 and GAPDH detection shown in Fig. 6 A. Left (21 % O2) and right (3 % O2) panels: After the protein transfer, the nitrocellulose membrane was cut in two pieces, below the 50 kDa marker, and each strip was incubated with antibodies against GAPDH or BiP/GRP78. Exposure times were: GAPDH 1 min (left and right); BiP/GRP78 1 min (left and right). [file 12885_2023_11271_MOESM9_ESM.pdf]
