# Supplementary material for: Targeting sphingolipid metabolism with the sphingosine kinase inhibitor SKI-II overcomes hypoxia-induced chemotherapy resistance in glioblastoma cells: effects on cell death, self-renewal, and invasion
Source: BMC Cancer. 2023 Aug 16;23:762. doi: 10.1186/s12885-023-11271-w (PMC10433583; doi:10.1186/s12885-023-11271-w)

Additional File 10 - Full-length blots of BiP/GRP78 and GAPDH detection shown in Figure 6 C.

After the protein transfer, the nitrocellulose membrane was cut in two pieces, between the 60 kDa and the 50 kDa markers and each strip was incubated with antibodies against GAPDH or BiP/GRP78. Exposure times were: GAPDH 20 sec; BiP/GRP78 1 min.

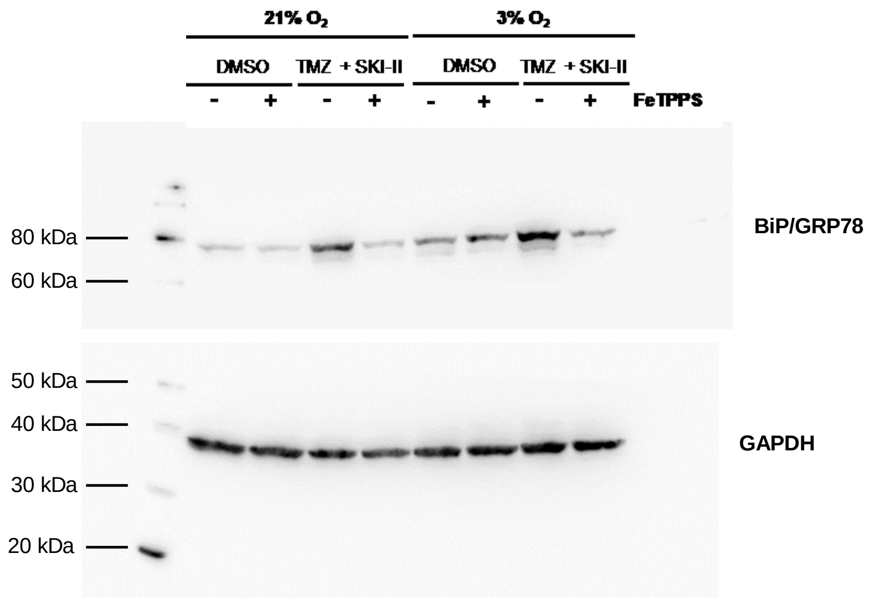

Supplement: Supplementary file 10 — Additional file 10. Full-length blots of BiP/GRP78 and GAPDH detection shown in Fig.6 C. After the protein transfer, the nitrocellulose membrane was cut in two pieces, between the 60 kDa and the 50 kDa markers and each strip was incubated with antibodies against GAPDH or BiP/GRP78. Exposure times were: GAPDH 20 sec; BiP/GRP78 1 min. [file 12885_2023_11271_MOESM10_ESM.pdf]
