# Supplementary material for: Targeting sphingolipid metabolism with the sphingosine kinase inhibitor SKI-II overcomes hypoxia-induced chemotherapy resistance in glioblastoma cells: effects on cell death, self-renewal, and invasion
Source: BMC Cancer. 2023 Aug 16;23:762. doi: 10.1186/s12885-023-11271-w (PMC10433583; doi:10.1186/s12885-023-11271-w)

Additional File 11

ELDA analysis: Full data files

Analysis of DMSO-1080 cells

Treatment at 21% O_2

n = 3

```
The value of the confidence choice entered was "0.95"

The value of the observed choice is        "FALSE"

The value of the test_unit_slope choice is "TRUE"

The value of the test_difference choice is "TRUE"
```

Limiting dilution data entered.

Counter	Dose	Tested	Response	Group
1	10	6	6.0	DMSO
2	5	12	11.7	DMSO
3	2.5	18	12.3	DMSO
4	1.25	18	7.3	DMSO
5	0.625	12	3.3	DMSO
6	0.3125	6	0.0	DMSO
7	10	6	5.3	TMZ
8	5	12	7.3	TMZ
9	2.5	18	9.0	TMZ
10	1.25	18	3.7	TMZ
11	0.625	12	1.3	TMZ
12	0.3125	6	0.7	TMZ
13	10	6	5.3	SKI
14	5	12	6.0	SKI
15	2.5	18	7.7	SKI
16	1.25	18	4.3	SKI
17	0.625	12	2.0	SKI
18	0.3125	6	0.3	SKI
19	10	6	3.3	D2
20	5	12	6.0	D2
21	2.5	18	6.0	D2
22	1.25	18	3.0	D2
23	0.625	12	1.3	D2
24	0.3125	6	0.3	D2

The number of lines of data entered = 24

Confidence intervals for 1/(stem cell frequency)

Group	Lower	Estimate	Upper
D2	11.89	7.53	4.77
DMSO	2.95	2.08	1.46
SKI	7.69	5.12	3.40
TMZ	6.79	4.57	3.07

Overall test for differences in stem

cell frequencies between any of the groups

Chisq	DF	P.value
21.8	3	7.34e-05

Pairwise tests for differences in stem cell frequencies

Group 1	Group 2	Chisq	DF	Pr(>Chisq)
D2	DMSO	20.7	1	5.32e-06
D2	SKI	1.60	1	0.206
D2	TMZ	2.74	1	0.0978
DMSO	SKI	11.0	1	0.000922
DMSO	TMZ	8.54	1	0.00348
SKI	TMZ	0.155	1	0.694

Goodness of fit tests. These test whether the log-dose slope equals 1. Rejection of the tests may be due either to batch effects (heterogeneity in the stem cell frequencies or assay success rate) or to a failure of the stem cell hypothesis.
Estimated slope is 0.965

Test	Chisq	DF	P Value
Likelihood ratio test of single-hit model	0.08	1	0.777
Score test of heterogeneity	0.240	1	0.624

Download PDF Version of the Image

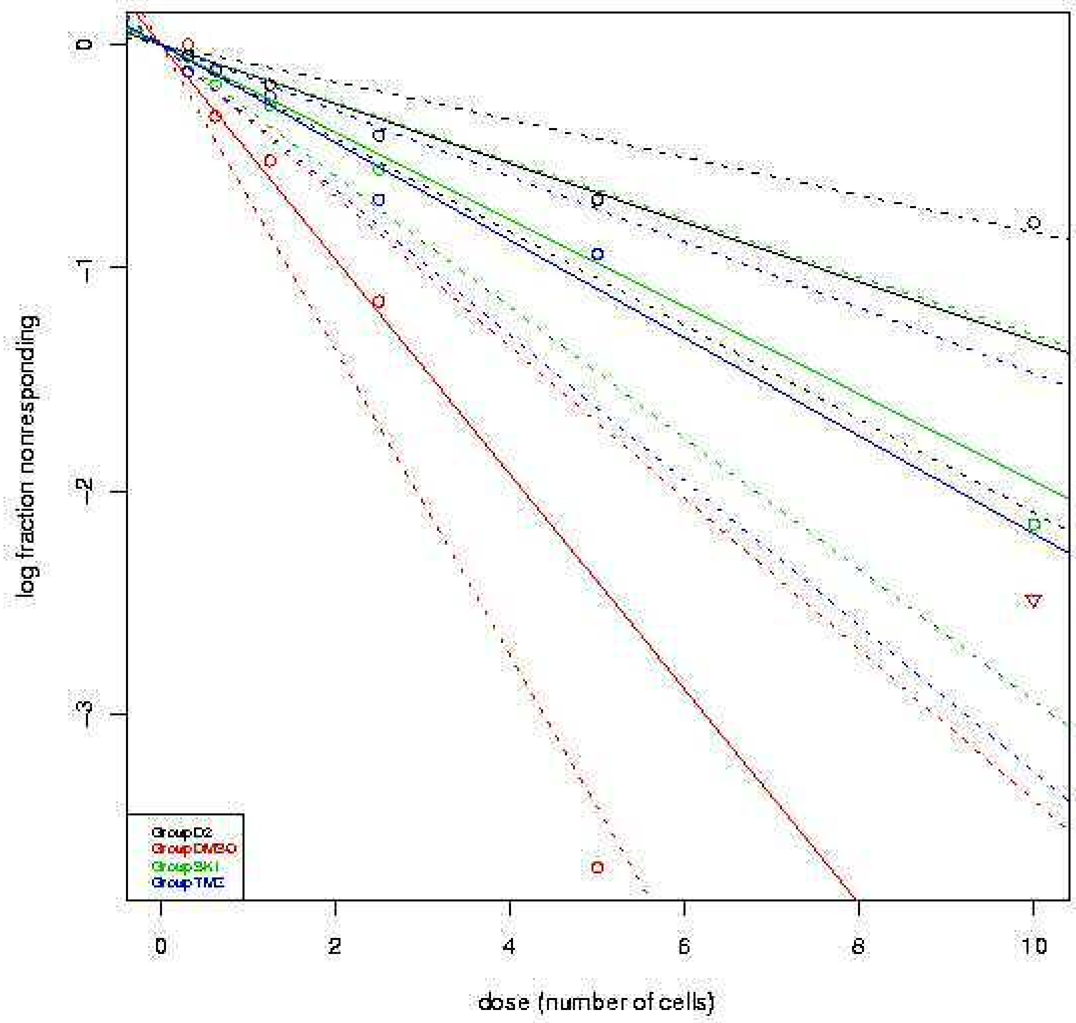

Analysis of DMSO-1080 cells

Treatment at 3 % O_2

n = 2

```
The value of the confidence choice entered was "0.95"

The value of the observed choice is        "FALSE"

The value of the test_unit_slope choice is "TRUE"

The value of the test_difference choice is "TRUE"
```

Limiting dilution data entered.

Counter	Dose	Tested	Response	Group
1	10	6	6	DMSO
2	5	6	4.5	DMSO
3	2.5	12	7	DMSO
4	1.25	12	3.5	DMSO
5	0.625	9	1.5	DMSO
6	0.3125	6	0	DMSO
7	10	6	5.5	TMZ
8	5	9	6.5	TMZ
9	2.5	14.5	7	TMZ
10	1.25	14.5	3	TMZ
11	0.625	8.5	1	TMZ
12	0.3125	5.5	0	TMZ
13	10	6	5	SKI
14	5	8	2	SKI
15	2.5	13.5	1	SKI
16	1.25	13	2.5	SKI
17	0.625	9	1	SKI
18	0.3125	6	0	SKI
19	10	6	4	D2
20	5	8.5	3	D2
21	2.5	15	1.5	D2
22	1.25	15	0.5	D2
23	0.625	8.5	1	D2
24	0.3125	5.5	0	D2

The number of lines of data entered = 24

Confidence intervals for 1/(stem cell frequency)

Group	Lower	Estimate	Upper
D2	24.60	13.28	7.16
DMSO	5.05	3.21	2.04
SKI	18.62	10.43	5.84
TMZ	6.55	4.25	2.76

Overall test for differences in stem cell

frequencies between any of the groups

Chisq	DF	P.value
20.2	3	0.000154

Pairwise tests for differences in stem cell frequencies

Group 1	Group 2	Chisq	DF	Pr(>Chisq)
D2	DMSO	14.1	1	0.000176
D2	SKI	0.303	1	0.582
D2	TMZ	9.37	1	0.00221
DMSO	SKI	10.2	1	0.00142
DMSO	TMZ	0.748	1	0.387
SKI	TMZ	6.13	1	0.0133

Goodness of fit tests. These test whether the log-dose slope equals 1. Rejection of the tests may be due either to batch effects (heterogeneity in the stem cell frequencies or assay success rate) or to a failure of the stem cell hypothesis.
Estimated slope is 1.19

Test	Chisq	DF	P Value
Likelihood ratio test of single-hit model	1.41	1	0.235
Score test of heterogeneity	1.67	1	0.196

Download PDF Version of the Image

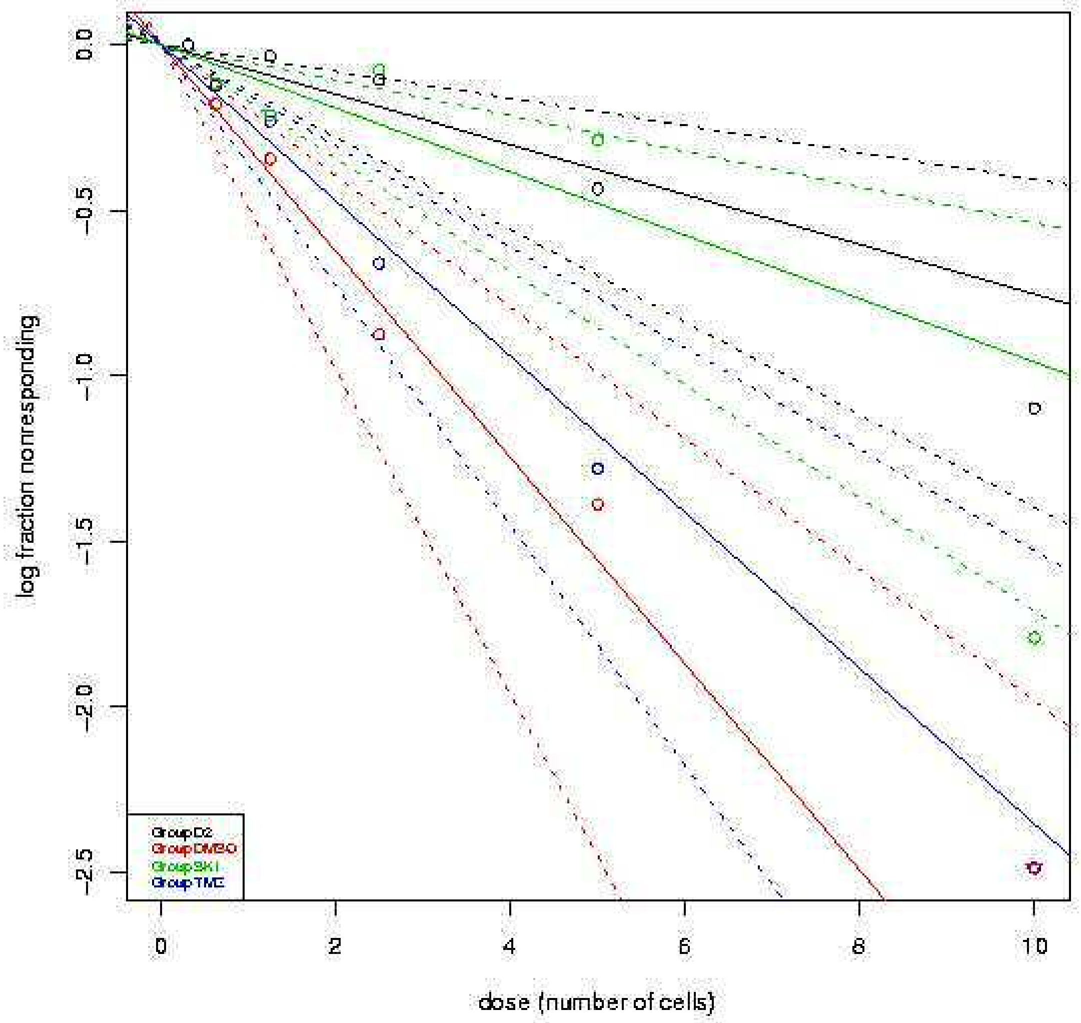

Analysis of TMZ-1080 cells

Treatment at 21% O_2

n = 3

```
The value of the confidence choice entered was "0.95"

The value of the observed choice is        "FALSE"

The value of the test_unit_slope choice is "TRUE"

The value of the test_difference choice is "TRUE"
```

Limiting dilution data entered.

Counter	Dose	Tested	Response	Group
1	10	6	6.0	DMSO
2	5	12	11.3	DMSO
3	2.5	18	11.3	DMSO
4	1.25	18	6.3	DMSO
5	0.625	12	2.0	DMSO
6	0.3125	6	0.3	DMSO
7	10	6	6.0	TMZ
8	5	12	11.3	TMZ
9	2.5	18	11.0	TMZ
10	1.25	18	8.0	TMZ
11	0.625	12	2.0	TMZ
12	0.3125	6	1.0	TMZ
13	10	6	4.0	SKI
14	5	12	4.0	SKI
15	2.5	18	3.0	SKI
16	1.25	18	1.3	SKI
17	0.625	12	1.0	SKI
18	0.3125	6	0.0	SKI
19	10	6	4.7	D2
20	5	12	3.7	D2
21	2.5	18	4.0	D2
22	1.25	18	1.0	D2
23	0.625	12	0.0	D2
24	0.3125	6	0.0	D2

The number of lines of data entered = 24

Confidence intervals for 1/(stem cell frequency)

Group	Lower	Estimate	Upper
D2	19.60	11.51	6.76
DMSO	3.54	2.47	1.73
SKI	20.41	11.91	6.95
TMZ	3.24	2.28	1.60

Overall test for differences in stem

cell frequencies between any of the groups

Chisq	DF	P.value
53.3	3	1.61e-11

Pairwise tests for differences in stem cell frequencies

Group 1	Group 2	Chisq	DF	Pr(>Chisq)
D2	DMSO	24.3	1	8.2e-07
D2	SKI	0.00742	1	0.931
D2	TMZ	27.7	1	1.41e-07
DMSO	SKI	25.4	1	4.58e-07
DMSO	TMZ	0.102	1	0.749
SKI	TMZ	28.9	1	7.5e-08

Goodness of fit tests. These test whether the log-dose slope equals 1. Rejection of the tests may be due either to batch effects (heterogeneity in the stem cell frequencies or assay success rate) or to a failure of the stem cell hypothesis.
Estimated slope is 1.27

Test	Chisq	DF	P Value
Likelihood ratio test of single-hit model	3.08	1	0.0791
Score test of heterogeneity	2.76	1	0.0966

Download PDF Version of the Image

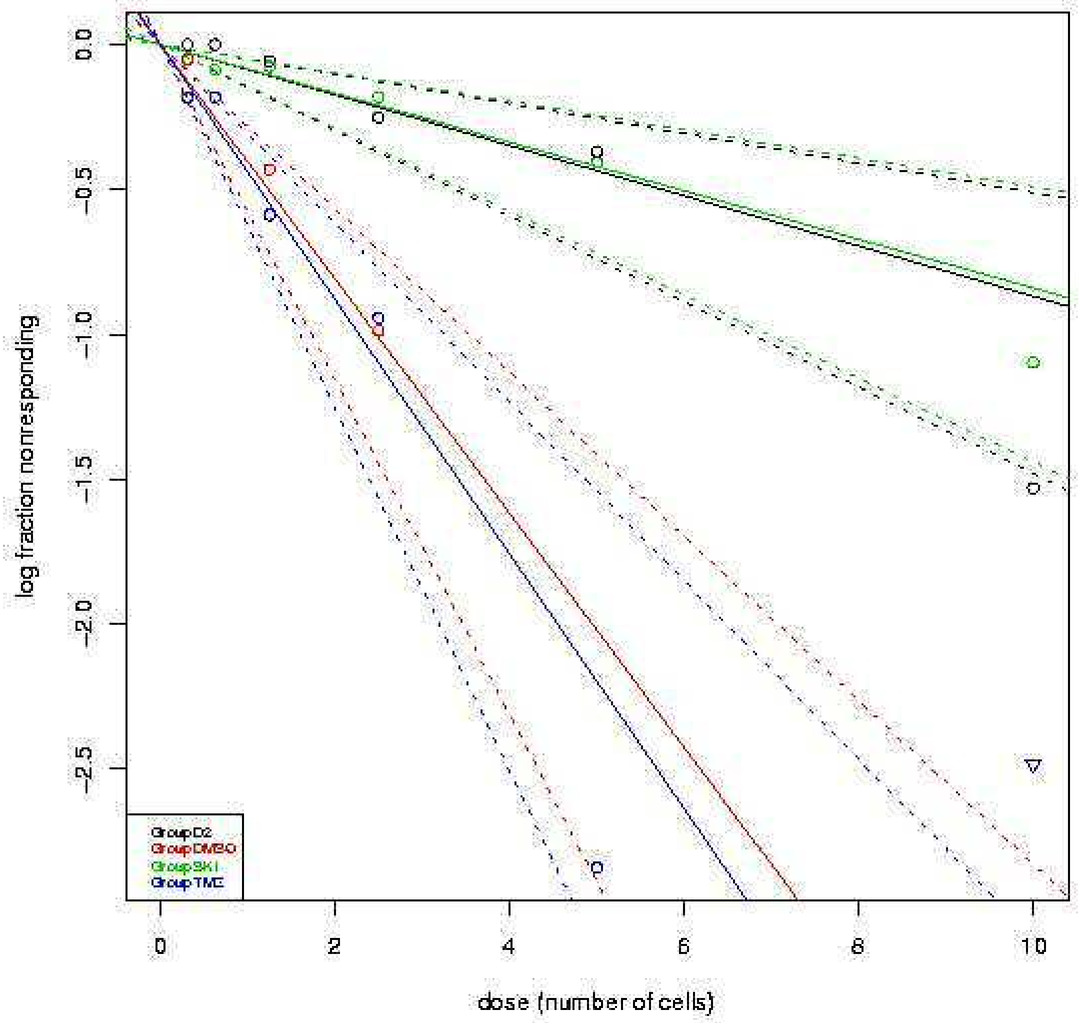

Analysis of TMZ-1080 cells

Treatment at 3 % O_2

n = 2

```
The value of the confidence choice entered was "0.95"

The value of the observed choice is        "FALSE"

The value of the test_unit_slope choice is "TRUE"

The value of the test_difference choice is "TRUE"
```

Limiting dilution data entered.

Counter	Dose	Tested	Response	Group
1	10	5.5	4.5	DMSO
2	5	8.5	5	DMSO
3	2.5	16.5	8.5	DMSO
4	1.25	17.5	2.5	DMSO
5	0.625	14	1	DMSO
6	0.3125	8	0.5	DMSO
7	10	5.5	5.5	TMZ
8	5	11	8.5	TMZ
9	2.5	16	6	TMZ
10	1.25	17.5	1.5	TMZ
11	0.625	14	1.5	TMZ
12	0.3125	8.5	0	TMZ
13	10	3.5	1.5	SKI
14	5	8	1	SKI
15	2.5	14	1	SKI
16	1.25	17.5	2	SKI
17	0.625	14	0	SKI
18	0.3125	8.5	0	SKI
19	10	5.5	0.5	D2
20	5	11.5	1	D2
21	2.5	16	0.5	D2
22	1.25	18	0	D2
23	0.625	15	0	D2
24	0.3125	8.5	0	D2

The number of lines of data entered = 24

Confidence intervals for 1/(stem cell frequency)

Group	Lower	Estimate	Upper
D2	361.96	90.67	22.71
DMSO	8.05	5.21	3.37
SKI	54.63	23.66	10.25
TMZ	7.42	4.90	3.23

Overall test for differences in stem

cell frequencies
between any of the
groups

Chisq	DF	P.value
44.7	3	1.06e-09

Pairwise tests for differences in stem cell frequencies

Group 1	Group 2	Chisq	DF	Pr(>Chisq)
D2	DMSO	29.3	1	6.2e-08
D2	SKI	3.06	1	0.0803
D2	TMZ	31.2	1	2.31e-08
DMSO	SKI	12.4	1	0.000423
DMSO	TMZ	0.0394	1	0.843
SKI	TMZ	13.7	1	0.000216

Goodness of fit tests. These test whether the log-dose slope equals 1. Rejection of the tests may be due either to batch effects (heterogeneity in the stem cell frequencies or assay success rate) or to a failure of the stem cell hypothesis.
Estimated slope is 1.26

Test	Chisq	DF	P Value
Likelihood ratio test of single-hit model	2.20	1	0.138
Score test of heterogeneity	1.18	1	0.278

Download PDF Version of the Image

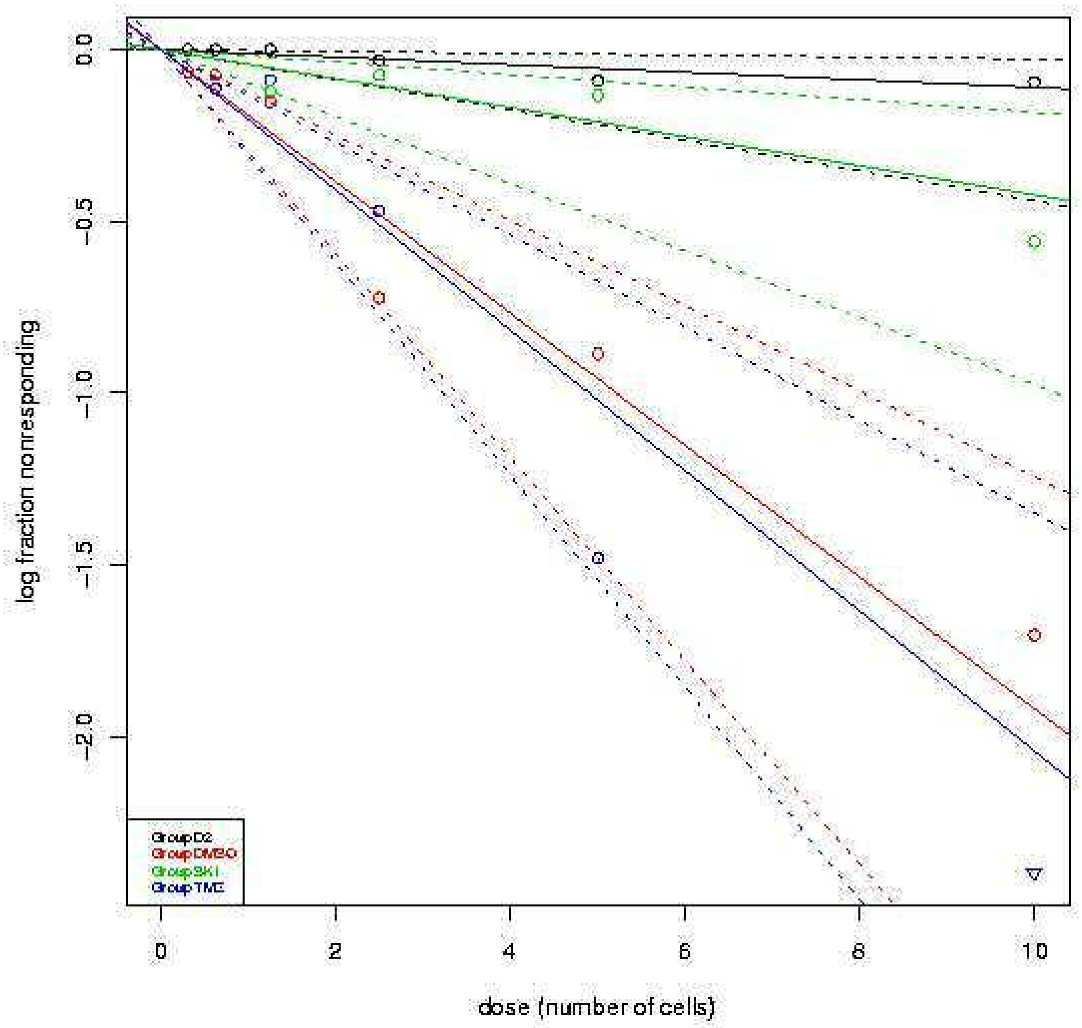

Supplement: Supplementary file 11 — Additional file 11. ELDA analysis: Full data files. [file 12885_2023_11271_MOESM11_ESM.pdf]
