# Supplementary material for: Targeting sphingolipid metabolism with the sphingosine kinase inhibitor SKI-II overcomes hypoxia-induced chemotherapy resistance in glioblastoma cells: effects on cell death, self-renewal, and invasion
Source: BMC Cancer. 2023 Aug 16;23:762. doi: 10.1186/s12885-023-11271-w (PMC10433583; doi:10.1186/s12885-023-11271-w)

Additional File 12 - Immunofluorescence detection of activated caspase-3 in treated GSC. DMSO-1080 and TMZ-1080 GSC cells plated on ornithin-coated glass coverslips were treated with 2.66 µM SKI-II, 48 µM temozolomide (TMZ) and the combination (TMZ+SKI-II) in triplicates. After 5 days of incubation, cells were fixed with 4% paraformaldehyde and stained for cleaved caspase-3, nestin and Dapi. (A) Quantification of cleaved caspase-3 positive cells. Data are presented as percent of total cells counted in 7 to 9 regions of triplicates, and for a total number of cells of 700 to 1100. (B) Representative images of apoptotic cells in 1080-TMZ cells treated with SKI-II under normoxia (left) or hypoxia (right). Magnification 40X, scale bar 50 µM. Left panels in each composite picture: enlarged images of apoptotic cells (marked by arrows in the right panels) manifesting morphological hallmarks of apoptosis such as chromatin condensation and cytoplasmic disintegration. n = 1

A

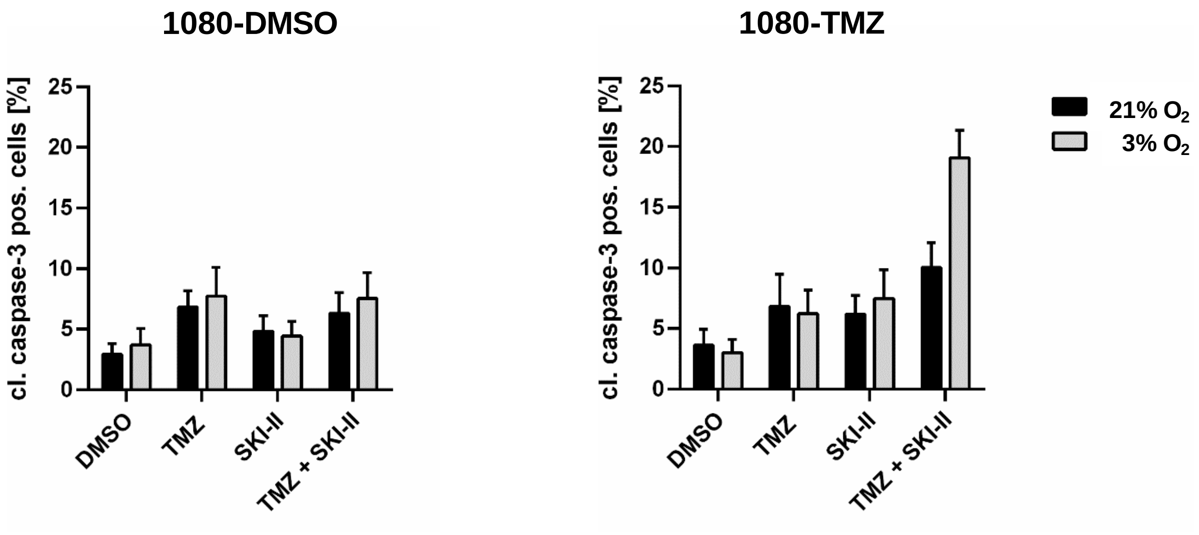

B

1080-TMZ cells, SKI-II, 21 % O_2 **1080-TMZ cells, SKI-II, 3 % O_2**

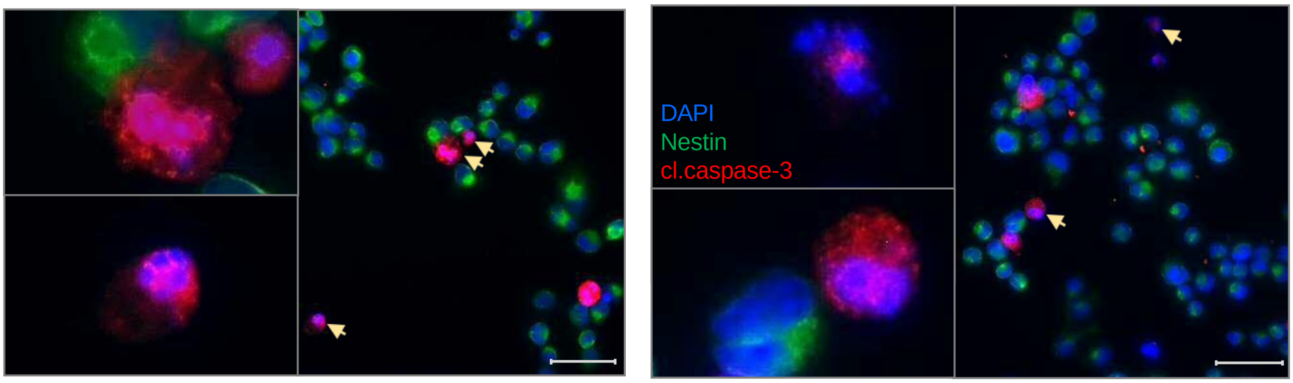

Supplement: Supplementary file 12 — Additional file 12. Immunofluorescence detection of activated caspase-3 in treated GSC. DMSO-1080 and TMZ-1080 GSC cells plated on ornithin-coated glass coverslips were treated with 2.66 µM SKI-II, 48 µM temozolomide (TMZ) and the combination (TMZ+SKI-II) in triplicates. After 5 days of incubation, cells were fixed with 4% paraformaldehyde and stained for cleaved caspase-3, nestin and Dapi. (A) Quantification of cleaved caspase-3 positive cells. Data are presented as percent of total cells counted in 7 to 9 regions of triplicates, and for a total number of cells of 700 to 1100. (B) Representative images of apoptotic cells in 1080-TMZ cells treated with SKI-II under normoxia (left) or hypoxia (right). Magnification 40X, scale bar 50 µM. Left panels in each composite picture: enlarged images of apoptotic cells (marked by arrows in the right panels) manifesting morphological hallmarks of apoptosis such as chromatin condensation and cytoplasmic disintegration. n = 1. [file 12885_2023_11271_MOESM12_ESM.pdf]
