# Supplementary material for: Targeting sphingolipid metabolism with the sphingosine kinase inhibitor SKI-II overcomes hypoxia-induced chemotherapy resistance in glioblastoma cells: effects on cell death, self-renewal, and invasion
Source: BMC Cancer. 2023 Aug 16;23:762. doi: 10.1186/s12885-023-11271-w (PMC10433583; doi:10.1186/s12885-023-11271-w)
Supplement: Supplementary file 13 — Additional file 13. The U3054 cells express the O6-methylguanine-DNA methyltransferase. [file 12885_2023_11271_MOESM13_ESM.pdf]

Additional file 13 - The U3054 cells express the O6-methylguanine-DNA methyltransferase

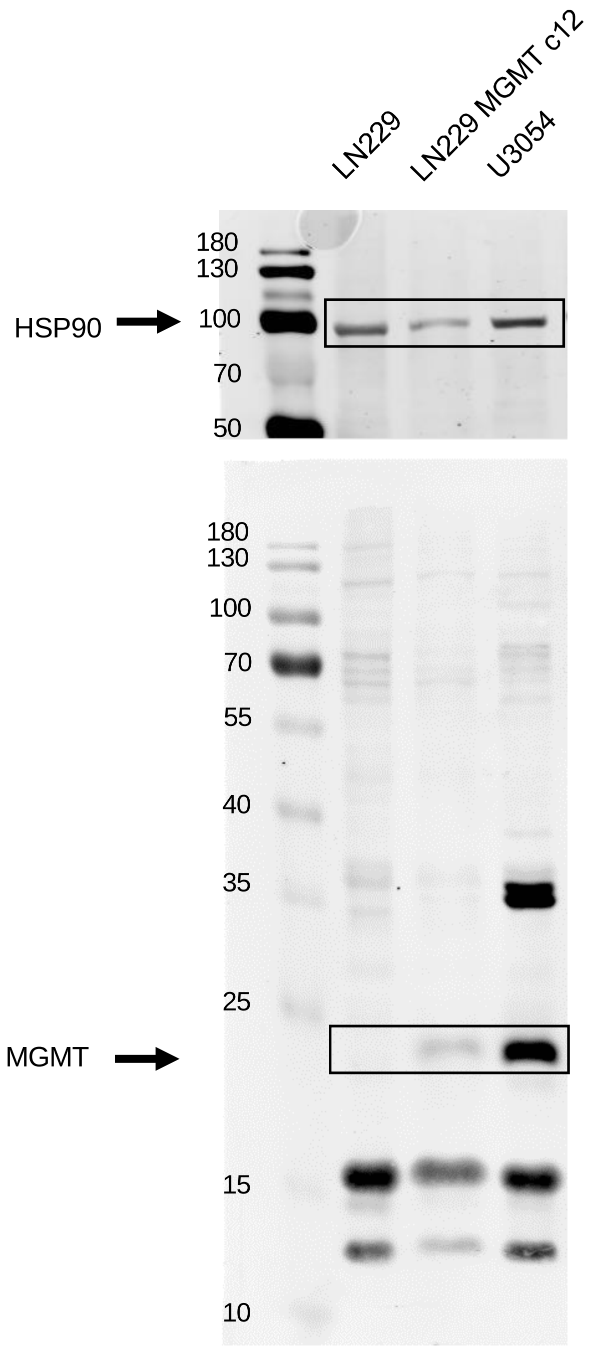

Western blot analysis of the O6-methylguanine-DNA methyltransferase (MGMT) in LN229 (negative control), LN229 MGMT c12 (positive control; DOI: 10.1371/journal.pone.0055665) and U3054 cells was performed as follows. Whole cell protein was extracted using the Invitrogen cell-extraction buffer (Invitrogen) according to the manufacturer's protocol. Protein concentrations were determined with the Bradford Protein Assay. Cell lysates were mixed with RotiLoad 1 (Carl Roth GmbH + Co. KG), boiled for 5 min at 90°C and stored at -20°C until use. For protein detection, 30 µg protein were loaded onto a 10 % polyacrylamide gel. Separated proteins were transferred onto a nitrocellulose membrane using a mini-protean blotting chamber. The membrane was blocked in 5% BSA-0.1% Tween 20-Tris buffer for 1h at room temperature. MGMT and HSP90 were sequentially detected on the same membrane using the Odyssey 9120 Infrared Imaging System (Li-Cor Biosciences). Primary antibodies were HSP90 (Santa Cruz; 1:1000; rabbit) and MGMT (Bernd Kaina, Medical Faculty of the Johannes Gutenberg-University Mainz; 1:750; mouse). Secondary antibodies were IRDye 800CW anti-mouse and anti-rabbit IgG (Li-Cor Inc, 1:5000). MGMT signal was detected at an intensity of 5.0; HSP90 signal at an intensity of 6.0 in the respective channels of the Odyssey 9120. Detection of HSP90 was run from the top of the membrane (180 kDa) up to the 50 kDa marker; detection of MGMT was run from top (180kDa) to bottom (10kDa).
